# Selection of suitable reference genes for qRT-PCR normalisation under different experimental conditions in *Eucommia ulmoides* Oliv

**DOI:** 10.1038/s41598-018-33342-w

**Published:** 2018-10-09

**Authors:** Jing Ye, Cang-Fu Jin, Nan Li, Min-Hao Liu, Zhao-Xue Fei, Li-Zheng Dong, Long Li, Zhou-Qi Li

**Affiliations:** College of Forestry, Northwest A&F University, Shaanxi, China

## Abstract

Normalisation of data, by choosing the appropriate reference genes, is fundamental for obtaining reliable results in quantitative real-time PCR (qPCR). This study evaluated the expression stability of 11 candidate reference genes with different varieties, developmental periods, tissues, and abiotic stresses by using four statistical algorithms: geNorm, NormFinder, BestKeeper, and RefFinder. The results indicated that ubiquitin-conjugating enzyme S (*UBC*) and ubiquitin-conjugating enzyme E2 (*UBC E*2) could be used as reference genes for different *E. ulmoides* varieties and tissues, *UBC* and histone H4 (*HIS4*) for different developmental periods, beta-tubulin (*TUB*) and *UBC* for cold treatment, ubiquitin extension protein (*UBA80*) and *HIS4* for drought treatment, and ubiquitin-60S ribosomal protein L40 (*UBA5*2) and *UBC E2* for salinity treatment. *UBC* and *UBC E2* for the group “Natural growth” and “Total”, *UBA80* and *UBC* for the group “Abiotic stresses”. To validate the suitability of the selected reference genes in this study, mevalonate kinase (*MK*), phenylalanine ammonia-lyase (*PAL*), and 4-coumarate-CoA ligase (*4CL*) gene expression patterns were analysed. When the most unstable reference genes were used for normalisation, the expression patterns had significant biases compared with the optimum reference gene combinations. These results will be beneficial for more accurate quantification of gene expression levels in *E. ulmoides*.

## Introduction

Gene expression analysis is an important part of molecular biology research. Quantitative real time polymerase chain reaction (qRT-PCR) has become a popular technology for studying gene expression patterns^1,2^. Absolute and relative quantification are two methods of presenting quantitative gene expression. The absolute quantification is done by comparing the quantification cycle (Cq) value of the sample with the standard curve^3^. As for the relative quantification, the qPCR data of target genes requires reference genes for calibration^4^. since the copy number of genes usually does not have any biological significance, researchers are more concerned about differential expression in gene analysis. Therefore, relative quantification has become the main method of gene expression analysis^5^.

In order to improve the reliability and accuracy of gene expression quantification, the standardisation of gene expression data is necessary. Calibration can eliminate system errors associated with the experimental process (i.e. the errors of sample quantification or between samples)^6,7^. Generally, the most common method for normalising data in gene expression experiments is to use reference genes as internal controls. Ideally, the reference gene expression profiles are not influenced by experimental conditions, however, a reference gene with universally stable expression under all experimental conditions (different varieties, tissues or organs, developmental periods, under biological or abiotic stresses etc.) has not yet been discovered. Therefore, screening and validating appropriate reference genes for different experimental conditions are critical for target gene expression data normalisation^8–10^. Usually, genes associated with maintaining basic cell functions (primary metabolism or cell structure) are selected as candidate reference genes^11–13^, such as 18S ribosomal RNA (*18S RNA*), actin (*ACT*), actin 97 (*ACT97*), histone H2B (*HIS2B*), histone H4 (*HIS4*), alpha-tubulin (*TUA*), beta-tubulin (*TUB*), ubiquitin-60S ribosomal protein L40 (*UBA52*), ubiquitin extension protein (*UBA80*), ubiquitin-conjugating enzyme S (*UBC*), and ubiquitin-conjugating enzyme E2 (*UBC E2*).

*Eucommia ulmoides* Oliver, the only member of the *Eucommiaceae* family (also called the Chinese rubber tree), is a unique economic tree species to China^14^. A highly valued traditional Chinese medicine is produced from its bark^15–19^ and it is also famous as a source of gutta-percha^20–24^. Therefore, studying the molecular basis of the economic traits and physiological patterns of *E. ulmoides* is of great importance in promoting the breeding process and improving use of active ingredients of *E. ulmoides*. Illustration of the expression levels of key genes is important, for example, in different varieties/genotypes, developmental periods, tissues, and so on. Chen *et al*.^25^ have selected housekeeping genes for transgene expression analysis in *E. ulmoides*. However, to date there have been no systematic analyses of reference gene screening in *E. ulmoides* for different varieties, developmental periods, tissues, and abiotic stresses.

*Trans*-polyisoprene rubber (Eu-rubber) and chlorogenic acid (CGA) are very important active ingredients of *E. ulmoides*. Studies on the expression patterns of *trans*-polyisoprene rubber biosynthesis and CGA biosynthesis genes play very important roles in *E. ulmoides* research. Mevalonate kinase (*MK*) is a key enzyme-coding gene related to *trans*-polyisoprene biosynthesis^26^; phenylalanine ammonia-lyase (*PAL*) and 4-coumarate-CoA ligase (*4CL*) are the key genes for the biosynthesis of CGA^27^. Their expression levels may be directly related to the contents of Eu-rubber and CGA.

In this study, 11 commonly used reference genes (*18S rRNA*, *ACT*, *ACT97*, *HIS2B*, *HIS4*, *TUA*, *TUB*, *UBA52*, *UBA80*, *UBC*, and *UBC E2*) were selected to evaluate expression stability in different varieties, tissues, leaf blade developmental periods and environmental conditions in *E. ulmoides*. Four different statistical software programs (geNorm^28^, NormFinder^29^, BestKeeper^30^, and RefFinder^31^) were used to analyse the stability of the candidate reference genes and select the most appropriate ones. This study will lay a foundation for future gene expression pattern research in *E. ulmoides*.

## Results

### Selection of reference genes, amplification specificity and efficiency, and cloning

Based on the *E. ulmoides* transcriptome data, we cloned 11 candidate reference genes (*18S rRNA*, *ACT*, *ACT97*, *HIS2B*, *HIS4*, *TUA*, *TUB*, *UBA52*, *UBA80*, *UBC*, and *UBC E2*) from “Huazhong4”. The sequences of these genes and the primers used for cloning are shown in Supplementary Figs S1–S11 and Supplementary Table S1. The primer pairs of all candidate reference genes and target genes were designed for qRT-PCR, and the amplicon lengths were controlled between 59 and 200 bp. Agarose gel electrophoresis (Supplementary Fig. S12) and melting curve analysis (Supplementary Fig. S13) were used to determine primer specificity. The amplification efficiency of qRT-PCR across all 11 reference genes varied from 89.1 to 106.4%, with *R*^*2*^ varying from 0.991 to 0.997 (Table 1 and Supplementary Fig. S14).

**Table 1:** Candidate reference genes and target genes description, primer sequences, and amplicon characteristics in this study.

Gene symbol	Gene description	Accession number	Primer sequence (5′-3′)	Size (bp)	PCR efficiency (%)	Regression Coefficient (R^2^)	Tm (°C)
**Reference genes**							
*18S rRNA*	18S ribosomal RNA	MH890464	CCCCGACTGTTCCTGTTAAT	59	105.5	0.995	77.5
			TGCGTTTCTATTTTGTTGGTTT				
*ACT*	actin	MH890466	GTGTTATGGTTGGGATGGG	108	100.2	0.996	80
			TGCTGACTATGCCGTGTTC				
*ACT97*	actin 97	MH890465	CGGGCAGGTCATCACTATCGG	200	97.7	0.997	86.9
			CGGCAATCCCAGGAAACATCG				
*HIS2B*	Histone H2B	MH890468	GGAAGAAATTGCCAAAGGATG	106	89.1	0.996	81.8
			TGCTTGAGGACCTTGAAGATGTA				
*HIS4*	histone H4	MH890467	GGGACAACATCCAGGGAATC	160	99.7	0.995	87
			GCGTGCTCGGTGTAGGTGA				
*TUA*	alpha-tubulin	MH890463	CATTTCCTCTTTGACTGCCTCC	185	102.8	0.992	84
			ATGCGGTGTTGGTGATTTCG				
*TUB*	beta-tubulin	MH890469	AAATGAGCACCAAGGAGGTG	119	100.6	0.991	82.6
			GGTTGGAGGAATATCGCAGA				
*UBA52*	ubiquitin-60S ribosomal protein L40	MH890471	GGCCAGGAAATACAACCAAG	120	90.4	0.995	84
			TTCTTCGGCCTCAACTGATT				
*UBA80*	ubiquitin extension protein	MH890472	GACCTACACCAAGCCGAAGA	114	105.9	0.992	83.9
			CACTCCTTCCTCAGCCTCTG				
*UBC*	ubiquitin conjugating enzyme S	MH890470	AGTGGGTGGTGCTGTAGTCC	121	102.5	0.992	82.7
			AACTCCCGTTTCGTTTGTTG				
*UBC E2*	ubiquitin-conjugating enzyme E2	MH890473	CAGTGGAGCCCTGCCCTTACC	110	106.4	0.995	82
			GCGATCTCTGGCACCAACGGG				
**Target genes**							
*MK*	mevalonate kinase	MH890474	GCCGATGAATCACAGAAA	181	105.3	0.993	82.5
			GCAACGGTGGTGGTAGTA				
*PAL*	phenylalanine ammonia-lyase	MH890475	CGGTTTGCCGTCGAATCTGT	74	107.6	0.99	82.5
			TCGCTATCTCCGCCCCCTTA				
*4CL*	4-coumarate-CoA ligase	MH890476	CGGTGCCTCTGAATCTGCT	143	106.4	0.997	85.5
			GATGTGGTGCTCTGCGTGC				

### Expression profile of the reference genes

**Figure 1:**
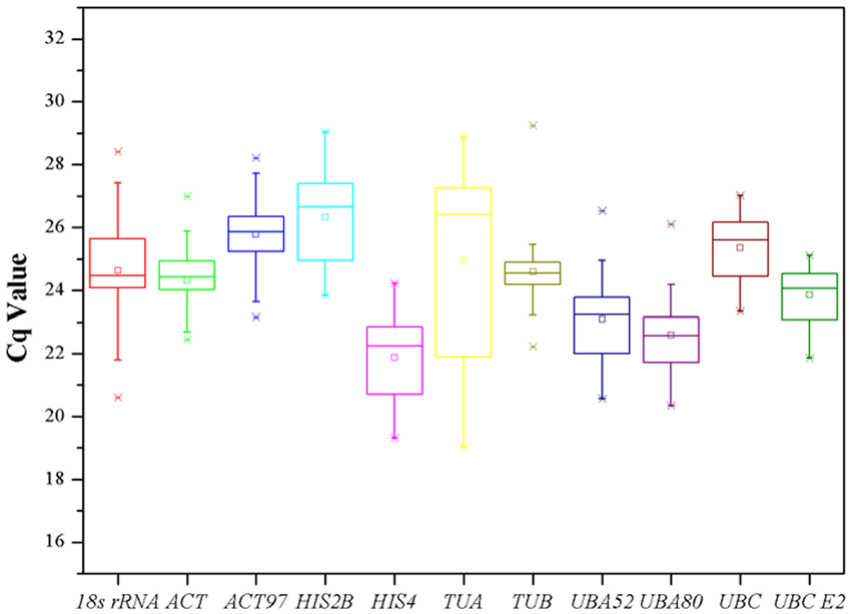
Cq values distribution of eleven candidate reference genes across all experimental samples of *E. ulmoides*. The outside box is determined from 25^th^ to 75^th^ percentiles, and the inside box represents the mean value. The line across the box is the median. The whiskers represent percentiles from 5^th^ to 95^th^, and asterisks represents outliers.

Cq values were used to quantify the expression levels of candidate reference genes; lower Cq values mean higher expression levels. The raw Cq values for all samples in this study were listed in Supplementary Table S2 (there were no Cq values in the negative controls), and a box and whiskers plot was used to describe the raw Cq value distribution (Fig. 1 and Supplementary Table S3). The 11 candidate reference genes had a wide expression range across all samples in this study (19.03 ≤ Cq ≤ 29.24). The results indicated that there were four genes (*HIS4*, *UBA52*, *UBA80*, *UBC E2*) with average Cq values between 21 and 24 cycles that displayed high expression levels; the other seven genes (*18S rRNA*, *ACT*, *ACT97*, *HIS2B*, *TUA*, *TUB*, *UBC*), which presented Cq values between 24 and 27, display intermediate expression levels. *HIS4* was the most expressed gene among the 11 candidate genes (mean Cq of 21.88). On the other hand, *HIS2B* was the least expressed gene (mean Cq of 26.32). In addition, every reference gene had different coefficients of variation (lower values represent less variability) in this study, as shown in Fig. 1; *TUA* had the most variation whereas *TUB* had the least variation, which indicated that *TUA* was the most unstable gene and *TUB* was the most stable gene of all the reference genes.

### Expression stability analysis of reference genes

In this study, six experimental conditions were performed, including different *E. ulmoides* varieties, different developmental periods, different tissues, cold treatment, drought treatment and salinity treatment. Furthermore, these experimental conditions were sorted into three different groups: “Natural growth” (Varieties, Periods, Tissues), “Abiotic stresses” (Cold, Drought, Salinity) and “Total” (all experimental conditions). In order to select appropriate reference genes for these experimental conditions and groups, four software programs (geNorm^28^, NormFinder^29^, BestKeeper^30^, and RefFinder^31^) were used to evaluate the stability of the 11 candidate reference genes.

### GeNorm analysis

The geNorm analysis results were presented in Table 2. It showed that, under different experimental conditions, the results of the most stable reference genes were differential. For different varieties and developmental periods of *E. ulmoides*, *UBC* was the most stable gene with *M* values of 0.337 and 0.706 respectively. *18S rRNA* was the least stable gene with *M* values of 0.767 and 1.037 respectively. For different tissues, *HIS2B* (*M* = 1.265) was the most stable gene, while *18S rRNA* (*M* = 3.879) was the most variable gene. Under cold treatment, *ACT* (*M* = 0.499) was the most stable gene, whereas *UBA52* (*M* = 1.051) was the most variable gene. Under drought treatment, *UBA80* (*M* = 0.458) was the most stable gene, and *TUB* (*M* = 0.877) was the least stable gene. Under salinity treatment, *UBA52* (*M* = 0.385) was the most stable gene, while *18S rRNA* (*M* = 1.093) was the most variable gene. Overall, *UBC* was the most stable gene for all experimental condition subsets, with M values of 0.961, 0.579, and 1.005 for “Natural growth”, “Abiotic stress”, and “Total”, respectively. *18S rRNA* was the least stable gene for both “Natural growth” and “Abiotic stress” with *M* values of 2.468 and 0.925 respectively; *TUA* was the most variable gene for “Total” with an *M* value of 2.226.

**Table 2:** The stability ranking of candidate reference genes by geNorm, NormFinder, BestKeeper and RefFingder.

Treatments	Rank	geNorm		NormFinder		BestKeeper			RefFinder	
		Gene	Stability (*M*)	Gene	Stability	Gene	SD [±Cq]	CV [%Cq]	Gene	Stability
Varieties	1	*UBC*	0.337	*UBC*	0.002	*UBC E2*	0.14	0.61	*UBC*	1.32
	2	*UBC E2*	0.338	*UBC E2*	0.003	*UBC*	0.24	0.98	*UBC E2*	1.41
	3	*UBA80*	0.352	*ACT*	0.004	*UBA80*	0.25	1.17	*UBA80*	2.91
	4	*ACT*	0.353	*HIS2B*	0.007	*ACT97*	0.28	1.11	*ACT*	4.53
	5	*HIS2B*	0.378	*UBA80*	0.007	*ACT*	0.3	1.22	*HIS4*	5.32
	6	*HIS4*	0.464	*ACT97*	0.017	*HIS2B*	0.31	1.24	*TUB*	6.9
	7	*UBA52*	0.486	*HIS4*	0.018	*18S rRNA*	0.31	1.18	*ACT97*	7.09
	8	*TUA*	0.494	*TUB*	0.018	*TUB*	0.32	1.32	*HIS2B*	7.33
	9	*TUB*	0.522	*UBA52*	0.018	*HIS4*	0.37	1.82	*UBA52*	7.69
	10	*ACT97*	0.524	*TUA*	0.02	*UBA52*	0.39	1.82	*TUA*	8.49
	11	*18S rRNA*	0.767	*18S rRNA*	0.029	*TUA*	0.41	1.91	*18S rRNA*	11
Periods	1	*UBC*	0.706	*UBC*	0.011	*HIS4*	0.2	0.98	*UBC*	1.32
	2	*HIS4*	0.727	*HIS4*	0.013	*UBC*	0.42	1.68	*HIS4*	1.86
	3	*UBA80*	0.759	*UBA80*	0.019	*UBA80*	0.44	1.95	*UBC E2*	2.78
	4	*UBC E2*	0.821	*UBC E2*	0.022	*18S rRNA*	0.45	1.83	*UBA80*	4.12
	5	*TUB*	0.882	*HIS2B*	0.024	*UBC E2*	0.46	1.98	*ACT97*	4.92
	6	*ACT*	0.891	*TUB*	0.028	*TUB*	0.47	1.9	*HIS2B*	5.62
	7	*HIS2B*	0.936	*ACT*	0.029	*ACT97*	0.5	1.95	*TUB*	6.96
	8	*ACT97*	0.952	*ACT97*	0.034	*HIS2B*	0.54	2.2	*18S rRNA*	7.95
	9	*TUA*	0.984	*UBA52*	0.037	*ACT*	0.56	2.27	*UBA52*	8.11
	10	*UBA52*	1.03	*18S rRNA*	0.037	*TUA*	0.62	2.79	*ACT*	8.74
	11	*18S rRNA*	1.037	*TUA*	0.061	*UBA52*	0.85	3.7	*TUA*	11
Tissues	1	*HIS2B*	1.265	*UBC*	0.003	*UBC E2*	0.75	3.26	*UBC*	2.21
	2	*UBC*	1.276	*ACT97*	0.008	*UBC*	0.95	3.88	*UBC E2*	2.74
	3	*UBA80*	1.311	*HIS2B*	0.008	*18S rRNA*	0.95	2.17	*HIS2B*	2.91
	4	*UBC E2*	1.365	*UBC E2*	0.022	*ACT*	0.98	4.02	*UBA80*	3.2
	5	*ACT97*	1.388	*UBA80*	0.028	*ACT97*	1.11	4.37	*UBA52*	4.09
	6	*UBA52*	1.401	*HIS4*	0.038	*HIS4*	1.12	5.08	*ACT97*	4.95
	7	*TUA*	1.515	*UBA52*	0.043	*HIS2B*	1.45	5.58	*HIS4*	6.26
	8	*HIS4*	1.541	*ACT*	0.044	*UBA80*	1.77	7.88	*TUA*	6.42
	9	*ACT*	1.693	*TUB*	0.06	*UBA52*	1.84	8.03	*ACT*	6.82
	10	*TUB*	1.86	*TUA*	0.065	*TUA*	1.93	8.38	*TUB*	9.97
	11	*18S rRNA*	3.879	*18S rRNA*	0.107	*TUB*	2.23	9.03	*18S rRNA*	10.74
Cold	1	*ACT*	0.499	*ACT97*	0.007	*TUB*	0.13	0.51	*TUB*	1.19
	2	*TUB*	0.504	*UBC*	0.009	*UBC*	0.16	0.6	*UBC*	2.51
	3	*HIS4*	0.532	*TUA*	0.009	*UBC E2*	0.17	0.71	*ACT*	2.66
	4	*UBC*	0.533	*ACT*	0.01	*ACT97*	0.19	0.71	*UBC E2*	4.41
	5	*TUA*	0.546	*TUB*	0.018	*ACT*	0.29	1.18	*HIS4*	4.74
	6	*ACT97*	0.557	*UBC E2*	0.019	*TUA*	0.3	1.13	*ACT97*	5.09
	7	*UBC E2*	0.561	*HIS2B*	0.039	*UBA80*	0.4	1.74	*TUA*	5.18
	8	*UBA80*	0.602	*HIS4*	0.073	*HIS4*	0.43	1.92	*UBA80*	7.74
	9	*HIS2B*	0.88	*UBA80*	0.163	*UBA52*	0.51	2.15	*HIS2B*	9.24
	10	*18S rRNA*	1.042	*18S rRNA*	0.24	*HIS2B*	0.74	2.72	*18S rRNA*	10.24
	11	*UBA52*	1.051	*UBA52*	0.359	*18S rRNA*	0.79	3.26	*UBA52*	10.46
Drought	1	*UBA80*	0.458	*HIS4*	0	*UBC*	0.08	0.32	*UBA80*	1.97
	2	*HIS4*	0.462	*UBA80*	0.002	*HIS4*	0.15	0.64	*HIS4*	2.21
	3	*UBA52*	0.483	*UBC*	0.008	*UBA80*	0.2	0.88	*UBA52*	3.41
	4	*UBC*	0.495	*ACT97*	0.01	*18S rRNA*	0.24	0.99	*ACT97*	4.43
	5	*ACT97*	0.507	*UBA52*	0.011	*UBA52*	0.36	1.53	*HIS2B*	4.45
	6	*18S rRNA*	0.587	*18S rRNA*	0.016	*ACT97*	0.4	1.56	*UBC*	4.47
	7	*HIS2B*	0.617	*HIS2B*	0.018	*TUA*	0.42	1.57	*UBC E2*	5.03
	8	*UBC E2*	0.63	*UBC E2*	0.022	*HIS2B*	0.47	1.73	*18S rRNA*	6
	9	*ACT*	0.725	*TUA*	0.025	*TUB*	0.48	1.95	*ACT*	8.89
	10	*TUA*	0.757	*ACT*	0.028	*UBC E2*	0.48	1.99	*TUA*	9.15
	11	*TUB*	0.877	*TUB*	0.034	*ACT*	0.68	2.92	*TUB*	10.46
Salinity	1	*UBA52*	0.385	*HIS2B*	0.003	*ACT97*	0.19	0.71	*UBA52*	2.06
	2	*UBC*	0.4	*UBA52*	0.006	*ACT*	0.24	0.97	*UBC E2*	2.78
	3	*UBC E2*	0.418	*UBC*	0.008	*UBA52*	0.34	1.44	*UBC*	3.13
	4	*HIS2B*	0.424	*UBC E2*	0.009	*UBC*	0.35	1.32	*TUB*	3.64
	5	*TUB*	0.447	*TUB*	0.013	*UBC E2*	0.36	1.49	*HIS2B*	3.66
	6	*UBA80*	0.477	*ACT*	0.015	*UBA80*	0.37	1.6	*ACT97*	4.3
	7	*ACT97*	0.484	*ACT97*	0.015	*TUB*	0.39	1.58	*ACT*	5.26
	8	*ACT*	0.489	*UBA80*	0.016	*HIS4*	0.44	1.94	*UBA80*	6.45
	9	*HIS4*	0.558	*TUA*	0.02	*HIS2B*	0.48	1.75	*HIS4*	8.74
	10	*TUA*	0.652	*HIS4*	0.021	*TUA*	0.69	2.53	*TUA*	10
	11	*18S rRNA*	1.093	*18S rRNA*	0.049	*18S rRNA*	1.16	4.7	*18S rRNA*	11
Natural growth	1	*UBC*	0.961	*UBC*	0.009	*UBC E2*	0.5	2.15	*UBC*	1.19
	2	*UBA80*	0.989	*UBC E2*	0.017	*ACT*	0.6	2.44	*UBC E2*	1.57
	3	*UBC E2*	1.03	*ACT97*	0.022	*UBC*	0.6	2.45	*ACT97*	3.46
	4	*ACT97*	1.067	*UBA80*	0.025	*ACT97*	0.65	2.56	*UBA80*	3.6
	5	*HIS2B*	1.155	*HIS2B*	0.028	*HIS4*	0.75	3.56	*HIS2B*	5.73
	6	*UBA52*	1.168	*ACT*	0.029	*HIS2B*	0.79	3.15	*HIS4*	6.44
	7	*ACT*	1.19	*TUB*	0.035	*UBA80*	0.89	4.03	*ACT*	6.45
	8	*HIS4*	1.215	*UBA52*	0.038	*TUB*	1	4.06	*UBA52*	6.51
	9	*TUA*	1.22	*HIS4*	0.04	*TUA*	1.03	4.64	*TUB*	8.49
	10	*TUB*	1.241	*TUA*	0.041	*UBA52*	1.14	5.07	*TUA*	10.24
	11	*18S rRNA*	2.468	*18S rRNA*	0.099	*18S rRNA*	1.33	5.36	*18S rRNA*	10.74
Abiotic stresses	1	*UBC*	0.579	*UBC*	0.011	*UBA80*	0.32	1.4	*UBA80*	1.32
	2	*UBA80*	0.58	*UBA80*	0.012	*UBC*	0.34	1.29	*UBC*	1.86
	3	*HIS4*	0.638	*UBC E2*	0.016	*TUB*	0.34	1.39	*TUB*	3.03
	4	*UBC E2*	0.642	*ACT97*	0.016	*HIS4*	0.35	1.55	*HIS4*	3.46
	5	*ACT97*	0.65	*TUA*	0.017	*UBC E2*	0.36	1.46	*UBC E2*	4.47
	6	*TUB*	0.691	*HIS4*	0.017	*UBA52*	0.39	1.66	*ACT97*	5.69
	7	*TUA*	0.693	*HIS2B*	0.02	*ACT97*	0.42	1.6	*TUA*	6.96
	8	*HIS2B*	0.734	*TUB*	0.02	*TUA*	0.44	1.63	*UBA52*	7.9
	9	*UBA52*	0.758	*UBA52*	0.025	*HIS2B*	0.52	1.91	*HIS2B*	8.49
	10	*ACT*	0.828	*ACT*	0.03	*ACT*	0.62	2.55	*ACT*	10
	11	*18S rRNA*	0.925	*18S rRNA*	0.033	*18S rRNA*	0.65	2.65	*18S rRNA*	11
Total	1	*UBC*	1.005	*UBC*	0.008	*ACT*	0.61	2.5	*UBC*	1.57
	2	*UBA80*	1.034	*UBC E2*	0.013	*TUB*	0.66	2.7	*UBC E2*	2
	3	*UBC E2*	1.039	*UBA80*	0.017	*ACT97*	0.69	2.68	*UBA80*	3.41
	4	*ACT97*	1.08	*ACT97*	0.02	*UBC E2*	0.71	2.98	*ACT97*	3.72
	5	*UBA52*	1.161	*HIS2B*	0.028	*UBA80*	0.78	3.44	*ACT*	5.2
	6	*HIS4*	1.177	*UBA52*	0.029	*UBC*	0.81	3.18	*TUB*	5.66
	7	*HIS2B*	1.247	*HIS4*	0.033	*UBA52*	0.97	4.19	*UBA52*	5.69
	8	*TUB*	1.313	*TUB*	0.037	*18S rRNA*	1.01	4.09	*HIS4*	6.34
	9	*ACT*	1.418	*ACT*	0.046	*HIS4*	1.08	4.94	*HIS2B*	7.65
	10	*18S rRNA*	2.064	*18S rRNA*	0.079	*HIS2B*	1.32	5	*18S rRNA*	9.46
	11	*TUA*	2.226	*TUA*	0.086	*TUA*	2.55	10.26	*TUA*	11

*SD* [±Cq], standard deviation of the Cq; *CV* [%Cq], coefficient of variance expressed as a percentage of the Cq level.

**Figure 2:**
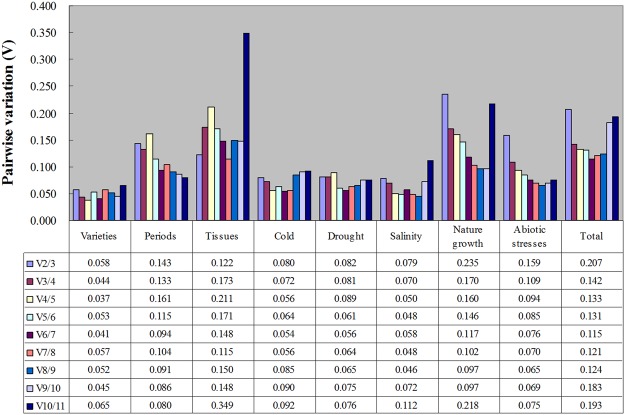
Pairwise variation (*V*) of candidate reference genes analyzed by geNorm. Pairwise variation (*V*_*n*_ */V*_*n+1*_) was analyzed between the normalization factors (NF_n_ and NF_n+1_) by geNorm to determine the optimal number of reference genes. The *V*_*n*_/*V*_*n+1*_ values below 0.15 suggested that there was no need to introduce another gene.

The optimal number of reference genes for normalisation depends on pair-wise variation (V_n/n+1_). When V_n/n+1_ < 0.15, it suggests that an extra reference gene is not necessary for normalisation. For six experimental conditions (Varieties, Periods, Tissues, Cold, Drought, Salinity), two reference genes were sufficient for accurate normalisation (Fig. 2); the most stable genes pairs for these conditions were *UBA80* and *UBC*, *UBC* and *UBCE2*, *UBA52* and *UBC80*, *TUB* and *UBC*, *HIS2B* and *UBC E2*, and *TUB* and *UBC E2*, respectively (Supplementary Fig. S4). For the group of “Abiotic stresses” and “Total”, V_2/3_ > 0.15 and V_3/4_ < 0.15 (Fig. 2). Therefore, the genes *UBC*, *UBC E2*, and *ACT97*, and *UBA80*, *UBC*, and *UBC E2* were chosen, respectively (Supplementary Fig. S15). However, for the “Natural growth” group, where V_5/6_ < 0.15 (Fig. 2), five reference genes were needed.

### NormFinder analysis

The stability value of each candidate reference gene was also analysed by NormFinder, wherein a lower stability value indicates higher expression stability. In this research, the results analysed by NormFinder were similar to the analysis by geNorm (Table 2). For different varieties, periods and tissues, *UBC* was the most stable reference gene. For cold treatment, *ACT97* was the most stable reference gene. For drought treatment, *HIS4* was the most stable reference gene. *HIS2B* was the most stable gene for salt treatment.

### BestKeeper analysis

The analysis results of BestKeeper are also shown in Table 2. The results indicated that all candidate reference genes were remarkably stable when expressed under most experimental conditions (different varieties, periods, cold treatment, and drought treatment) in this study. The rankings by BestKeeper analysis showed that the most stable reference genes were *UBC E2* (*CV* ± *SD* = 0.61 ± 0.14) and *UBC* (*CV* ± *SD* = 0.98 ± 0.24) for different varieties of *E. ulmoides*. *HIS4* (*CV* ± *SD* = 0.98 ± 0.20) and *UBC* (*CV* ± *SD* = 1.68 ± 0.42) were the most stable genes for leaf blade developmental periods. For different tissues, only four reference genes were expressed stably; the most stable genes were *UBC E2* (*CV* ± *SD* = 3.26 ± 0.75) and *UBC* (*CV* ± *SD* = 3.88 ± 0.95). *TUB* (*CV* ± *SD* = 0.51 ± 0.13) and *UBC* (*CV* ± *SD* = 0.60 ± 0.16) showed the most stable expression under cold treatment. *UBC* (*CV* ± *SD* = 0.32 ± 0.08) and *HIS4* (*CV* ± *SD* = 0.64 ± 0.15) were the best reference genes under drought treatment. Ten reference genes displayed significantly stable expression in the salinity treatment; *ACT97* (*CV* ± *SD* = 0.71 ± 0.19) and *ACT* (*CV* ± *SD* = 0.97 ± 0.24) were the most stable genes, while *18S rRNA* (*CV* ± *SD* = 4.70 ± 1.16) was considered not suitable for gene expression normalisation. Seven reference genes had remarkably stable expression in the “Natural growth” group, in which *UBC E2* (*CV* ± *SD* = 2.15 ± 0.50), *ACT* (*CV* ± *SD* = 2.44 ± 0.60), and *UBC* (*CV* ± *SD* = 2.45 ± 0.60) were the most stable genes (Table 2). For the “Abiotic stresses” group, all of the 11 reference genes were stably expressed; of these, *UBA80* (*CV* ± *SD* = 1.40 ± 0.32), *UBC* (*CV* ± *SD* = 1.29 ± 0.34), and *TUB* (*CV* ± *SD* = 1.39 ± 0.34) were the most stable genes (Table 2). For the “Total” group, seven reference genes presented remarkably stable expression, in which *ACT* (*CV* ± *SD* = 2.50 ± 0.61) and *TUB* (*CV* ± *SD* = 2.70 ± 0.66) were the most stable genes (Table 2).

### RefFinder analysis

We estimated the geomean of ranking values obtained from geNorm, NormFinder, and BestKeeper programs using RefFinder software. This allowed us to generate a recommended comprehensive ranking of reference genes for accurate transcript normalisation in each experimental set. The results indicated that *UBC* and *UBC E2* were the most stable genes for different varieties and tissues, that *UBC* and *HIS4* were the most stable genes for different development stages, that *TUB* and *UBC* were the most stable genes for cold treatment, that *UBA80* and *HIS4* were the most stable genes for drought treatment, and that *UBA52* and *UBC E2* were the most stable genes for salinity treatment. *UBC* and *UBC E2* were the most stable genes for the groups “Natural growth” and “Total”, and *UBA80* and *UBC* were the most stable genes for the group “Abiotic stresses” (Table 2).

### Reference gene validation

To validate the accuracy of selected reference genes, the relative expression levels of *MK*, *PAL*, and *4CL* were analysed in all the experimental conditions involved in this study. For each experiment condition, the two most stable and two unstable reference genes, according to RefFinder and the reference genes combination according to geNorm, were selected for normalisation.

**Figure 3:**
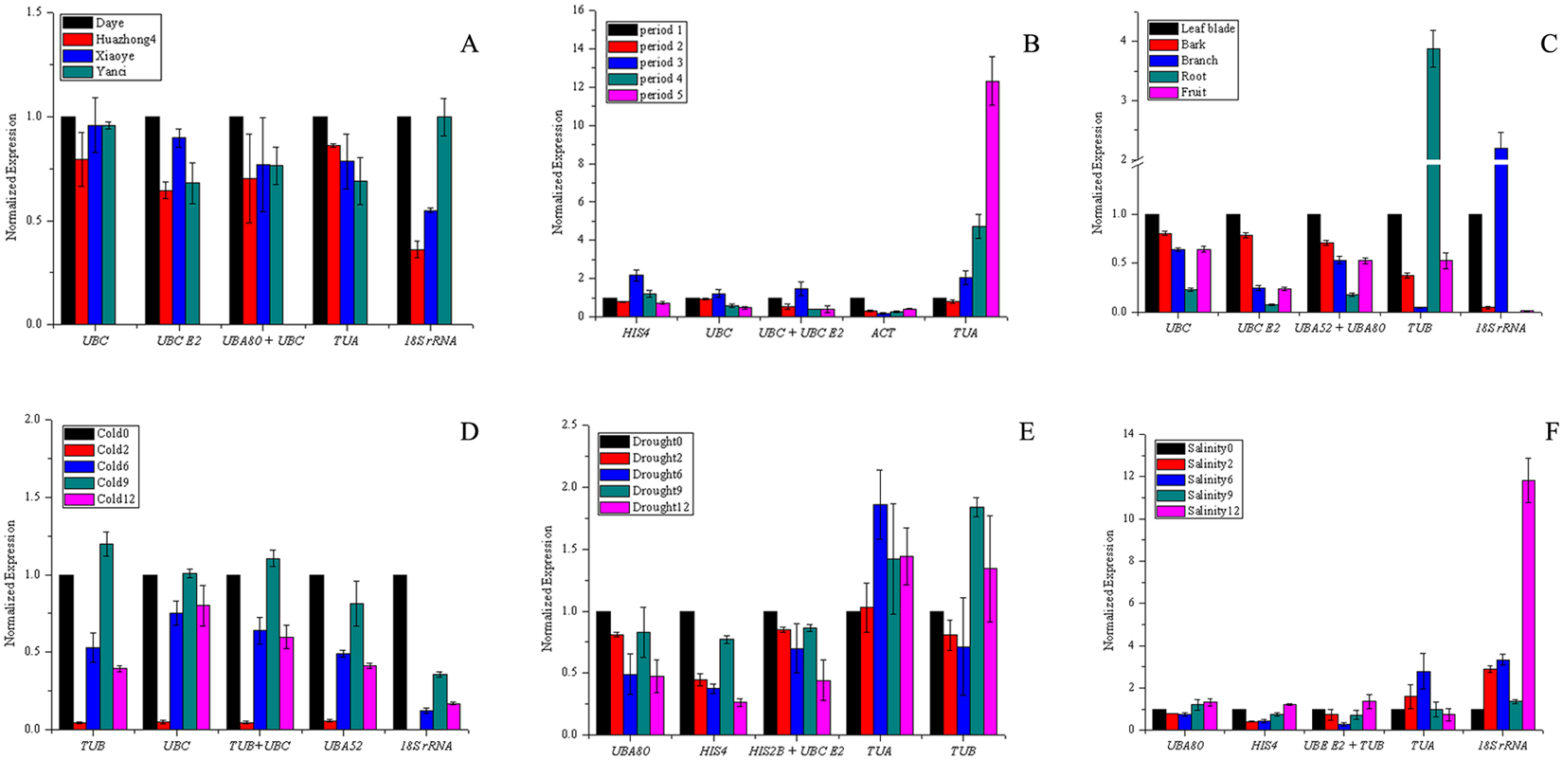
Effect of different reference genes to normalize the relative expression of *MK* gene. (**A**) Leaf blade samples of different varieties, (**B**) leaf blade samples at different periods, (**C**) different tissues, (**D**) cold treatment, (**E**) drought treatment, (**F**) salinity treatment.

Among the four varieties, there was no significant difference in the expression of *MK*. *MK* has the highest expression in “Daye”, followed by “Xiaoye” and “Yanci”; the lowest was “Huazhong4” (Fig. 3A). In the five leaf developmental stages, *MK* was up-regulated with approximately 1.8-fold changes in the third period and down-regulated in the second, fourth and fifth periods (Fig. 3B). Among the five tissues, *MK* has the highest expression in the leaves, followed by the bark (0.85-fold changes); the lowest was the root (0.2-fold changes) (Fig. 3C). Under cold treatment, *MK* was down-regulated at 2 h, 6 h, and 12 h, but especially at 2 h (0.05-fold changes) (Fig. 3D). Under drought treatment, the expression of *MK* was down-regulated, and the lowest expression levels were observed at 12 h (0.44-fold changes) (Fig. 3E). Under the salinity treatment, the expression levels of *MK* first decreased but then increased with treatment time. The lowest expression was at 6 hours after treatment (0.28- fold changes) (Fig. 3F).

**Figure 4:**
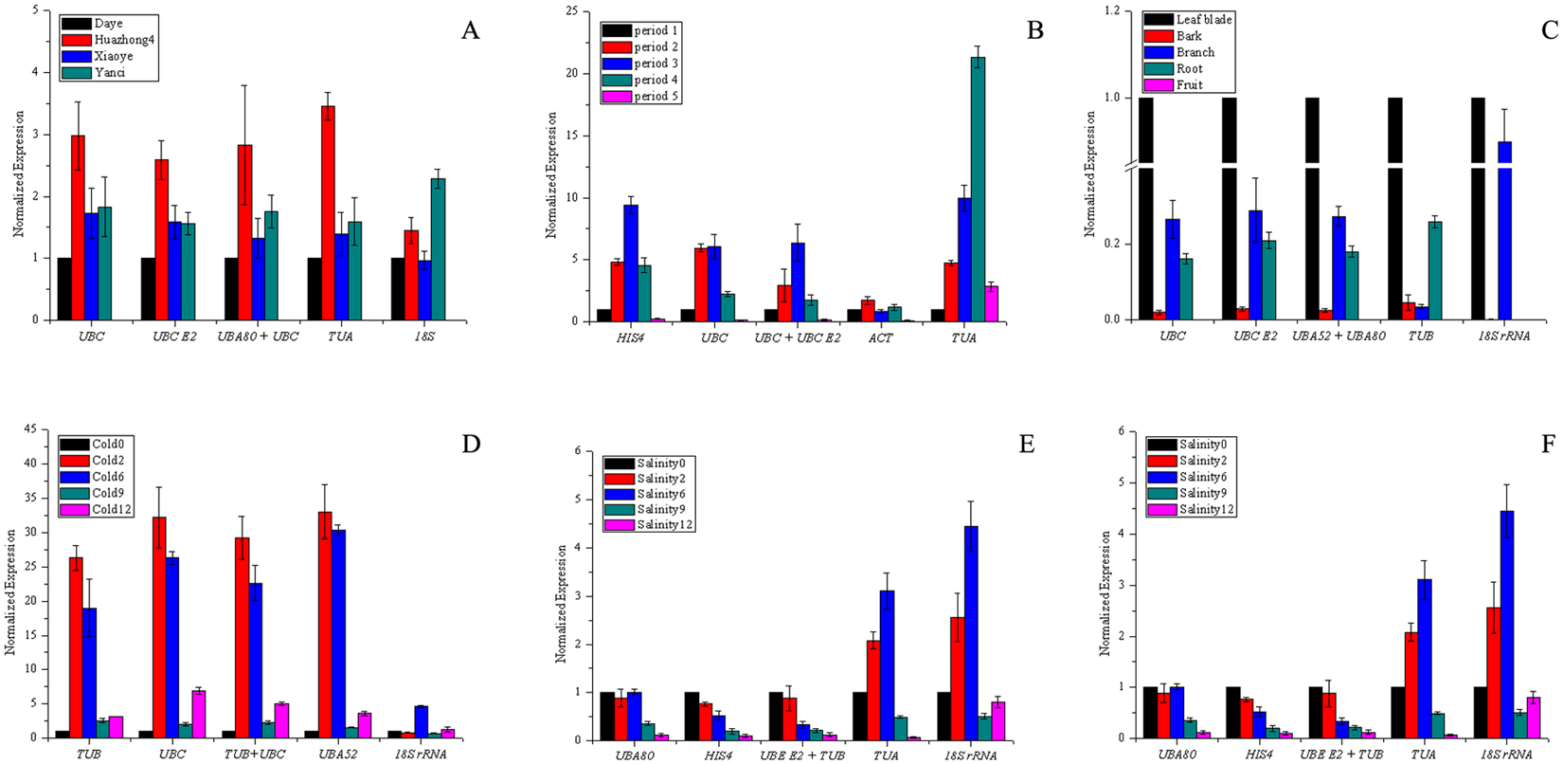
Effect of different reference genes to normalize the relative expression of *PAL* gene. (**A**) Leaf blade samples of different varieties, (**B**) leaf blade samples at different periods, (**C**) different tissues, (**D**) cold treatment, (**E**) drought treatment, (**F**) salinity treatment.

Among the four varieties, *PAL* has the highest expression in “Huazhong4”, followed by “Yanci”, and the lowest expression in “Daye” (Fig. 4A). During leaf development, the expression level of *PAL* first increased with leaf growth and then decreased, with the highest expression levels in period three (Fig. 4B). Among the five tissues, *PAL* has the highest expression in leaves, followed by branches, and was not expressed in fruits (Fig. 4C). Under cold treatment, the expression of *PAL* first increased sharply, then decreased with the prolongation of the treatment, but slightly increased 12 h after treatment (Fig. 4D). In the drought and salinity treatments, the expression levels of *PAL* decreased with the prolongation of treatment time (Fig. 4E, F).

**Figure 5:**
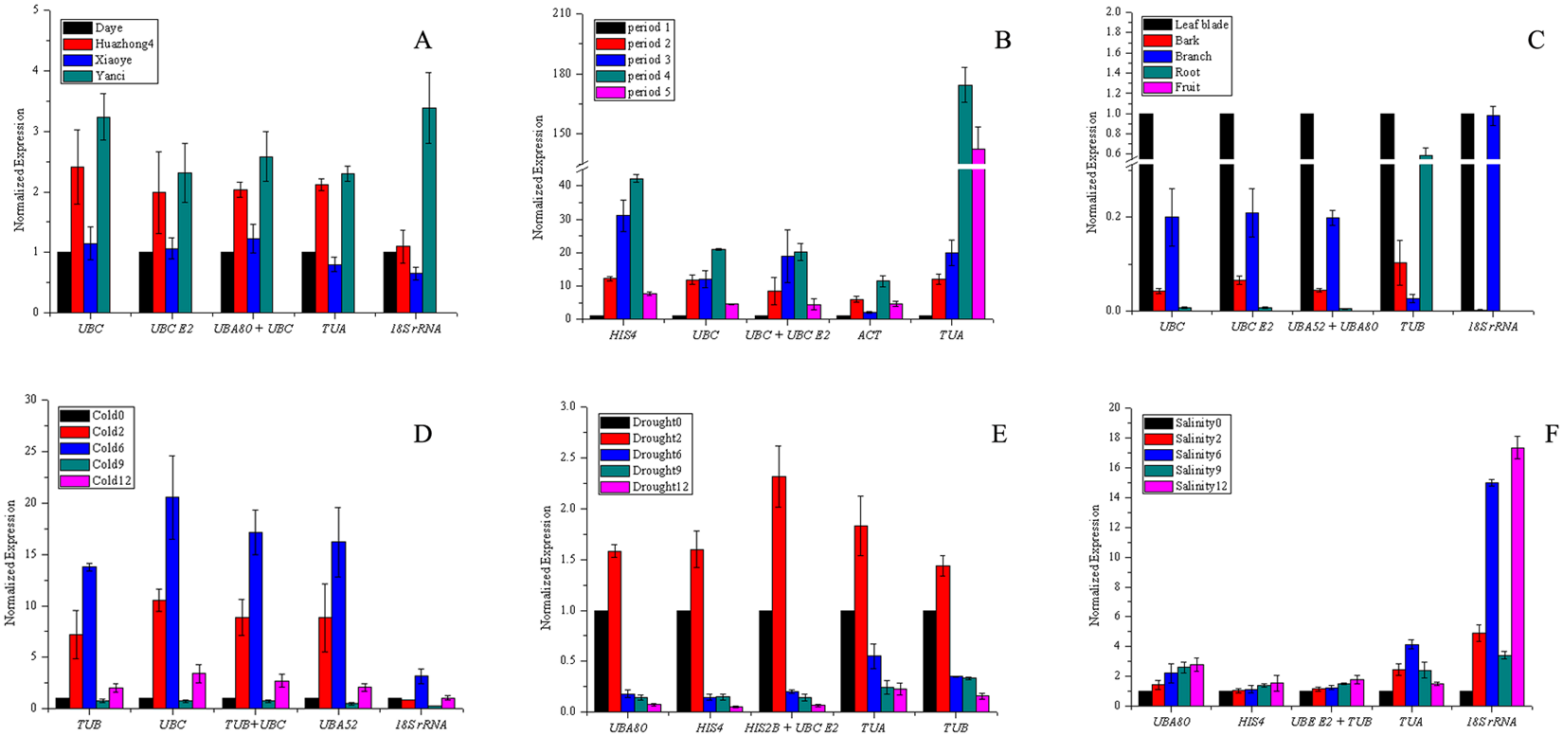
Effect of different reference genes to normalize the relative expression of *4CL* gene. (**A**) Leaf blade samples of different varieties, (**B**) leaf blade samples at different periods, (**C**) different tissues, (**D**) cold treatment, (**E**) drought treatment, (**F**) salinity treatment.

Among the four varieties, *4CL* has the highest expression in “Yanci”, followed by “Huazhong4”, and the lowest expression levels in “Daye” (Fig. 5A). During leaf development, the expression level of *4CL* increased with leaf development, but decreased in period five (Fig. 5B). In different tissues, the expression level of *4CL* was highest in leaves, followed by branches, but it was not expressed in fruits (Fig. 5C). In the first six hours of the cold treatment, the expression level of *4CL* increased with the prolongation of the treatment time, decreased sharply in the 9^th^ hour of treatment, and increased slightly in the 12^th^ hour of treatment (Fig. 5D). During drought treatment, the expression of *4CL* was up-regulated at first, but then decreased gradually with treatment time (Fig. 5E). The expression of *4CL* slowly increased as the salt treatment time was prolonged (Fig. 5F).

Our results confirm that using different reference genes for normalisation causes great differences among the expression patterns of *MK*, *PAL* and *4CL*.When the stable reference genes and optimum reference gene combinations were used for normalisation, the expression patterns of *MK*, *PAL* and *4CL* were similar. However, when the most unstable reference genes were used for normalisation, the expression patterns of *MK*, *PAL* and *4CL* had significant biases compared with the optimum reference gene combinations. The results illustrate that a stably expressed reference gene was essential to an accurate normalisation of target gene expression.

## Discussion

Gene expression pattern analysis in different experimental conditions is necessary for the functional analysis of genes^32^. Presently, many methods can be used to study gene expression levels, but qRT-PCR has become a powerful technology to research gene expression patterns because of its accuracy and sensitivity^33,34^. In qRT-PCR analysis, reference genes with stable expression levels and suitable expression abundance are preconditions that ensure the accuracy of gene expression analysis in different experimental conditions or species^35^. Ideal reference genes should be stably expressed in all experimental conditions. Many studies have emphasised that there is neither a universal reference gene nor a defined number of genes that should be used; thus, it is necessary to experiment in order to determine the appropriate reference gene or gene combination^36^. Reliable reference genes have been determined in many plant species under different cultivars, developmental stages, biotic stresses and abiotic stresses. For instance, selected suitable reference genes have been found for *Coffea arabica*^37^, peach^38^,carrot^39^, berry^40^, celery^41^, pepper^42^, maize^43^, and so on. However, to the best of our knowledge, the selection of reference genes has only been carried out in transgenic *E. ulmoides*. In this study, 11 commonly used reference genes (*18S rRNA*, *ACT*, *ACT97*, *HIS2B*, *HIS4*, *TUA*, *TUB*, *UBA52*, *UBA80*, *UBC* and *UBC E2*) were selected as candidate reference genes to analyse under three natural growth conditions and three abiotic stress conditions. All candidate reference genes used in this study presented a suitable expression abundance (19 < Cq < 29), which can further evaluate their expression stability. To date, this study is the first report of a systematic analysis of reference genes in different varieties, tissues, developmental stages and environmental conditions in *E. ulmoides*.

In order to avoid the one-sidedness of an algorithm for the analysis of the stability of reference genes, several statistical methods are usually simultaneously used to analyse the best reference genes in different experimental conditions^44,45^. In the present study, three commonly used statistical programs (geNorm, NormFinder, and BestKeeper) were employed to evaluate and determine suitable reference genes. Similar to other studies, different statistical methods produced different stability rankings in each experimental condition, but the results were roughly the same. As reported in other studies, the most discrepant results in the gene stability ranking were obtained with BestKeeper^36^. In this study, for the “Total” group, *UBC*, *UBA80*, and *UBC E2* were identified as the most stable genes by geNorm and NormFinder, BestKeeper showed *ACT* and *TUB* to be the best reference genes despite the fact that, *ACT* and *TUB* were ranked as the 8^th^ and 9^th^ genes by both geNorm and NormFinder. Therefore, it is very important for this study to use RefFinder to comprehensively analyse the results of geNorm, NormFinder and BestKeeper. The results of RefFinder are based on the geometric mean of the three software programs and the delta CT method to obtain the final ranking.

Using a single reference gene for normalisation will lead to deviations in the results^28,46^. Thus, two or more reference genes for standardization purposes will reduce the experimental error^47^. In the present study, geNorm was employed to determine the optimal number of reference genes for calibration in different experimental conditions. Our results showed that under different varieties, tissues, developmental stages and environmental conditions, the pair-wise variation was V_2/3_ < 0.15, which indicated that two reference genes were sufficient for optimal normalisation. But in the groups “Natural growth”, “Abiotic stresses” and “Total”, V_2/3_ > 0.15, which indicated that more genes were needed. However, although using multiple reference genes can make the results more accurate, it is not a required standard^28^.

The suitability of the selected reference genes has been assessed by analysing the expression levels in three target genes that related to *trans*-polyisoprene (Eu-rubber) biosynthesis (*MK*) and CGA biosynthesis (*PAL* and *4CL*). *MK* is a key enzyme-coding gene related to *trans*-polyisoprene biosynthesis^26^; *PAL* and *4CL* are the upstream key enzymes of CGA^27^. In our study, the expression levels of *MK*, *PAL* and *4CL* were different in different varieties, different tissues, different developmental stages and abiotic stresses. Indeed, the contents of Eu-rubber and CGA in different varieties, tissues and developmental stages of *E. ulmoides* were different. In addition, the expression levels of *MK*, *PAL* and *4CL* were largely different in cold, drought, and salinity treatments. This is possibly due to the fact that these abiotic stresses are related to the content of Eu-rubber and CGA.

Additionally, we used both the most stable and the most unstable reference genes for normalisation to compare with the optimal reference gene combination for normalisation, the results are quite different. When the stable reference genes and optimum reference gene combinations were used for normalisation, the expression patterns of *MK*, *PAL* and *4CL* were similar. However, when the most unstable reference genes were used for normalisation, the expression patterns of *MK*, *PAL* and *4CL* had significant biases compared with the optimum reference gene combinations. This indicates that the reference genes screened in this study are reliable.

The selected stable reference genes in this study will be beneficial for more accurate quantification of gene expression levels in *E. ulmoides* for different varieties, developmental stages, tissues and environmental conditions.

## Methods

### Plant materials and treatments

For non-stress treatments, the third leaves from the base of the *E. ulmoides* branches were collected on April 9^th^, 2016 to evaluate expression stability in four different varieties: “Xiaoye”, “Daye”, “Huazhong4” and “Yanci”. Leaves of “Huazhong4” at five developmental stages were collected every 10 days from March 31^st^ to May 9^th^, 2016 to evaluate expression stability in different leaf blade developmental periods. These five periods include leaves from germination to maturity; blade widths were 0.5 cm, 2.5 cm, 4 cm, 5.5 cm and 7.3 cm for each period, respectively. The third leaves from the base branches, barks from annual branches, one-year-old branches, and fibril roots of “Huazhong4” plants were harvested to evaluate expression stability in different tissues. All of the above materials were collected from the nursery of the College of Forestry, Northwest A & F University in Yangling, Shaanxi, China. For stress treatment, one- year-old potted plants of “Huazhong4”, kept in the natural environment, were carefully removed from soil, and the roots were gently washed by distilled water. For drought and salinity treatments, the plants were immersed in complete medium containing 15% PEG_6000_ and 200 mM NaCl, respectively, for 0, 2, 6, 9 and 12 h. For cold treatment, the plants were immersed in complete medium and were transferred at 4 °C for 0, 2, 6, 9 and 12 h. All treatments were performed in our laboratory. The leaf blade samples (the third leaves from the top of the plants) were separately collected and immediately frozen in liquid nitrogen, and then were stored at −80 °C. Each experimental condition had three biological replicates.

### RNA isolation and cDNA reverse transcription

Total RNA was extracted using the Plant RNA Kit (OMEGA, Omega Bio-Tek, Shanghai, China) and treated with RNase-free DNase I according to the manufacturer’s instructions. RNA concentration and purity were measured by the NanoDrop Nano-200 (All For Life Science, Hangzhou, Zhejiang, China), and RNA integrity was estimated by 1.2% agarose gel electrophoresis. cDNA (10 μL) was synthesised from 500 ng of total RNA using the PrimeScript™ RT reagent Kit (TaKaRa Biotech Co., Ltd., Dalian, China). Random 6 mers and the Oligo dT Primer were used together according to the manufacturer’s instructions.

### Candidate reference genes selection, primer design, and gene cloning

The sequences of 10 candidate reference genes (*18S rRNA*, *ACT*, *ACT97*, *HIS2B*, *HIS4*, *TUA*, *TUB*, *UBA52*, *UBA80*, and *UBC*) originated from our *E. ulmoides* transcriptome (not published), and the primer sequences of *UBC E2* originated from the study of Chen *et al*.^48^. The primers were designed by Primer Premier 5.0 software. The primer sequences of candidate reference genes used in this study were embodied in Table 1 and Supplementary Table S1. The primer specificities and amplicons size were verified by 4% agarose gel electrophoresis. A five-fold cDNA dilution series with three replicates per concentration was used to made standard curves for estimation of amplification efficiency (*E* = (10^[−1/slope]^ −1) × 100%) and the correlation coefficient (*R*^*2*^)^49^. The sequences of 11 candidate reference genes from *E. ulmoides* were cloned using 2 × Taq Plus Master Mix (Vazyme Biotech Co., Ltd., Nanjing, China) as the polymerase. The PCR reaction (50 μL) contained 25 μL of 2 × Taq Plus Master Mix, 19 μL of dd H_2_O, 2 μL of the template cDNA, and 2 μL of each primer (10 nmol·m L^−1^). The amplification conditions were as follows: 3 min at 94 °C for denaturation; 35 cycles of 30 s at 94 °C (denaturation), 30 s at 55 °C (annealing), and 60 s at 72 °C (extension); and a final step of 10 min at 72 °C for extension. PCR products were gel-purified, ligated into the pMD 19-T vector, and then transformed into *Escherichia coli*. The bacterial liquids were sequenced by Gen Script Corporation (Nanjing, China).

### Quantitative real-time PCR assay

qRT-PCR reactions were performed in a CFX96 Connect Real-time PCR Detection System (Bio-Rad Laboratories, Inc., Hercules, CA, USA) using SYBR Premix *Ex Taq* (TaKaRa Biotech Co., Ltd., Dalian, China). Each PCR reaction mixture (20 μL) contained 2 μL of diluted cDNA (20 × dilution), 10 μL of SYBR Green II Mix, 0.8 μL of each primer (10 nmol.mL^−1^), and 6.4 μL of ddH_2_O. The amplification conditions were as follows: 95 °C for 30 s to pre-denaturation, 40 cycles at 95 °C for 10 s to denaturation, and 58 °C for 20 s to annealing and extension. Melting curves were analysed from 60 °C to 95 °C to confirm primer specificity and lack of primer dimers. Each reaction was repeated three times. The negative controls were performed on each plate and for each sample, with ddH_2_O and total RNA to replace the cDNA.

### Data analysis

Cq values were obtained by setting the baseline threshold to a mean of 75.55. The raw Cq data are shown in Supplementary Table S2. Four widely used software: geNorm^28^, NormFinder^29^, BestKeeper^30^, and RefFinder^31^ were used to analyse the candidate reference gene’s expression stability. When using the geNorm and NormFinder algorithms for analyses, the raw Cq data needs to be transformed into relative quantities. However, when using the BestKeeper and RefFinder software, the Cq values need not to be converted.

GeNorm calculates the expression stability measure (*M*) and analyzes the pair-wise variation (V) for each candidate reference genes, then excludes the most unstable genes which with highest *M*-value progressively. In addition, pair-wise variation V_n_/V_n+1_ (0.15 recommended threshold), determines the optimal number of reference genes for normalization^36,41^.

NormFimder calculates the expression stability value (*SV*) on the basis of intra- and inter-group for each reference gene^29^. The high expression stability of this gene is reflected in a low *SV*-value.

BestKeeper calculates the stability of candidate reference genes based on standard deviation (*SD*), Pearson correlation coefficient (*r*), and coefficient of variation (*CV*) with the Cq data of all candidate genes. The most stable gene is with the lowest *SD* and *CV* values. The range of variation of *SD* should be below 1^36,41^.

RefFinder can generate a comprehensive ranking of candidate reference genes in each experimental condition^31^.

### Validation of reference genes

To validate the reliability of selected reference genes, two most stable and two unstable reference genes and optimum internal reference gene combinations were used to normalize the relative expression patterns of *MK*, *PAL*, *4CL* in each experimental condition. The relative expression levels were calculated by 2^−△△Ct^ method^5^.

## Electronic supplementary material

Supplementary Information
