# Supplementary material for: Selection of suitable reference genes for qRT-PCR normalisation under different experimental conditions in Eucommia ulmoides Oliv
Source: Sci Rep. 2018 Oct 9;8:15043. doi: 10.1038/s41598-018-33342-w (PMC6177395; doi:10.1038/s41598-018-33342-w)
Supplement: Supplementary file 1 — Supplementary Information [file 41598_2018_33342_MOESM1_ESM.pdf]

Selection of suitable reference genes for qRT-PCR normalisation under different experimental conditions in *Eucommia ulmoides* Oliv.

Jing Ye, Cang-Fu Jin, Nan Li, Min-Hao Liu, Zhao-Xue Fei, Li-Zheng Dong, Long Li, Zhou-Qi Li *

College of Forestry, Northwest A&F University, Shaanxi, China

*Correspondence: lizhouqi@nwsuaf.edu.cn

Figure S1. Nucleotide sequence of *TUA* from *E. ulmoides*.

GGAGCGTCTCTCTGTTGATTATGGTAAGAAATCAAAGTTGGGTTTCACTGTCTACCCCT
CCCCCCAGGTCTCTACATCAGTTGTTGAGCCCTACAACAGCGTCCTCTCAACTCACTCT
CTCCTGGAACACACGGATGTTGCTATCCTTCTTGACAATGAGGCCATTTACGATATCTGC
AGACGCTCACTTGACATTGAGCGGCCCACATACACCAACCTCAACCGTCTCGTCTCTC
AGGTGATTTCCTCCTTGACCGCATCTCTGAGGTTTGACGGTGCCCTGAATGTGGACATG
ACCGAATTCCAAACCAACTTGGTACCCTACCCAAGAATCCACTTCATGCTCTCCTCCTA
TGCTCCTGTCATCTCTGCTGAGAAAGCCTACCACGAGCAACTCTCTGTGGCCGAAATC
ACCAACAGTGCCTTTGAGCCTTCCTCCATGATGGCTAAATGTGACCCCCGTCACGGCA
AATACATGGCTTGCTGTCTCATGTACCGAGGCGATGTCGTCCCCAAAGACGTCAATGCC
GCCGTTGCCACCATCAAGACCAAACGGACCATCCAGTTCGTTGATTGGTGCCCCACTG
GTTTCAAGTGTGGTATTAATTACCAGCCGCCAACTGTTGTTCCGGGTGGTGATTTGGCT
CGGGTTCAGAGGGCGGTTTGTATGATCTCGAACTCGACAAGTGTGGCGGAGGTATTTT
CGAGGATTGACCATAAGTTTGATCTCATGTATGCGAAGCGGGCTTTTGTGCATTGGTAT
GTGGGTGAGGGAATGGAAGAAGGAGAGTTTTCGGAAGCTCGGGAGGATCTTGCAGCT
CTTGAGAAGGATTATGAGGAAGTTGGTGCCGAGGGGGCGGAGGATGAGGGTGATGAA
GGGGATGAGTACTGAGTTGGGTTTCTAGCAATATGGGCATTACTGTCTTATTGTTTCTGT
TTGTCTTTCGTTTTTGATTGTTTGTTTGGTGTTCGTACATTTGCTCTGTTTGAAAAAATG
TGTTGAAACTTTCGACTTCTGTTTTCAGCTATAAAGGTTAACTAATGCAAGTATTGTTTT
TTTTAATCTTTTAATCAAGATACACGAATGATCTAATTGTGAAAGGGAAACGCTTAATTT
ATTCTAGTTTTTCATTGATTATGATGATGGACCTTTCAACAATTCATTCACTGT

Figure S2. Nucleotide sequence of *18S rRNA* from *E. ulmoides.*

AAAAGCTCGTAGTTGAACCTTGGGCCTGGCTGGCCGGTCCGCCTCACCGCGTGTACTG
GTCCGGCCGGGCCTTTCCTTCTGGGGAACCTCATGCCCTTCACTGGGCGTGTTGGGGA
ACCAGGACTTTTACTTTGAAAAAATTAGAGTGTTCAAAGCAGGCCTTTGCTCGAATACA
TTAGCATGGAATAATAGAATAGGACGTGTGGTTCTATTTTGTTGGTTTCTAGGACCGCCG
TAATGATTAATAGGGATAGCCGGGGGCATTTGTATTCAATGGCAAGGGGTGAAATCCTT
GGATTCATTGAAGACAAACTTCTGCGAAAGCATTTGCCAAGGAGATCTTCATAAATCA
AGAACGAAGGTGAGGAGGTCGAAAACGAGTAGATACGGTGGTAGTCTTCACAGTAAA
CTAGGCAGAGGAGGGAGCGAGCG

Figure S3. Nucleotide sequence of *ACT97* from *E. ulmoides*.

AGATGATGCTCCACGGGCTGTGTTCCCAAGCATTGTAGGTCGACCACGCCACATAGGC
GTTATGGTCGGTATGGGCCAAAAAGATGCATATGTTGGAGATGAAGCTCAATCCAAACG
TGGTATATTGACACTCAAATATCCAATTGAACACGGCATTGTGAGCAATTGGGATGATAT
GGAAAAGATATGGCATCACACCTTCTACAACGAGCTTCGTGTTACTCCTGAAGAGCAC
CCCGTCCTCCTGACTGAAGCTCCGTTGAACCCGAAGGCCAATCGCGAGAAGATGACTC
AGATCATGTTCGAGACATTTAATACGCCTGCAATGTATGTTGCTATTCAAGCAGTTCTTT
CGCTGTATGCTAGTGGTCGAACCACCGGAATTGTTTTGGACTCTGGTGACGGTGTGAG
CCACACGGTACCCATATATGAAGGTTATGCCCTCCCACATGCCATCCTCCGTCTAGACTT
GGCTGGCCGCGACCTGACCGACCACCTCATGAAAATCCTGACCGAGCGTGGCTACTCC
TTCACCACCACCGCTGAGCGGGAAATTGTCCGCGACATGAAGGAAAAACTCGCCTACA
TTGCCCTAGACTATGAGCAAGAGATGGAGACCTCAAAGACAAGCTCCTCCGTCGAGA
AGAGCTACGAGCTGCCCGACGGGCAGGTCATCACTATCGGCGCCGAGCGGTTCCGATG
CCCTGAGGTTCTTTTCCAGCCGTCGGCAATTGGAATCGAGGCCGCCGGAATTCACGAG
ACGACATATAATTCGATCATGAAGTGTGATGTGGATATCAGGAAGGATTTGTATGGAAAT
ATTGTGTTGAGTGGTGGTTCAACGATGTTTCCTGGGATTGCCGAGAGGATGAATAAGG
AGATTACGGCGCTTGCACCACCTAGCAGCATGAAGATCAAGGTGGTGGCACCTCCTGA
GAGGAAGTACAGTGTCTGGATTGGAGGGTCTATCTTAGCTTCACTCAGTACCTTCCAGC
AGATGTGGATTTCAAAGGCCGAATATGATGAATCTGGTCCTTCCATTGTTCATCGTAAAT
GCTTCTGAGTTGTGTGAAGACAAACCCAATGGTATATCTTCCCTTTATTTTCGAAACCG
TGTCGTATTAGAGTTCTGTTTGG

Figure S4. Nucleotide sequence of *ACT* from *E. ulmoides.*

AATTATATGGGACCGTCAAACATGCCTTTCGATTTTTCCCTCGTATGTTGCCTAAATTTTT
TCCTCTAGGCTGGATTTGCTGGTGATGATGCTCCAAGGGCTGTGTTTCCCAGTATCGTT
GGCCGACCTCGACATACTGGTGTTATGGTTGGGATGGGCCAAAAGGATGCTTATGTTGG
TGATGAAGCACAGTCAAAAAGAGGTATCCTTACCTTGAAATACCCAATAGAACACGGC
ATAGTCAGCAATTGGGACGATATGGAGAAGATCTGGCACCACACTTTCTACAACGAAC
TTCGTGTTGCACCTGAAGAGCACCCCATACTACTTACAGAAGCTCCATTGAACCCCAA
GGCTAACAGAGAGAAAATGACCCAAATCATGTTCGAGACCTTTGATGCGCCTGCAATG
TATGTTGCTATTCAGGCCGTTCTGTCTCTTTATGCCAGTGGTCGTACAACAGGTATCGTC
TTGGACTCCGGTGACGGCGTCAGCCACACGGTACCAATTTACGAAGGTTATGCCCTTC
CCCATGCAATCCTCCGGCTAGACCTCGCTGGCCGTGATCTAACGGATTCCCTAATGAAA
ATCCTCACCGAAAGAGGTTACACCTTCACCACAACAGCAGAACGAGAAATCGTACGT
GACATCAAGGAGAAGCTCGCCTACATTGCCCTCGACTATGAGCAGGAACTGGAGACTG
CCAAGACCAGCTCCGCCATCGAGAAGAGCTACGAACTGCCTGACGGTCAGGTCATCA
CCATAGGGGCGGAGCGGTTCCGCTGTCCGGAAGTTCTGTTCCAGCCGTCATTCATCGG
GATGGAGTCGCCGGGGATTCATGAAACCACTTACAATTCGATCATGAAGTGTGACGTT
GATATCCGGAAGGATTTGTATGGGAATATTGTGCTTAGCGGTGGGTCAACGATGTTTCC
AGGGATTGCTGAGCGTATGAATAAGGAGATTTCGGCACTTGCCCCTAGCAGCATGAAG
ATCAAGGTGGTGGCACCTCCTGAGAGGAAGTACAGTGTCTGGATTGGAGGGTCTATCT
TAGCTTCACTCAGTACCTTCCAGCAGATGTGGATTTCAAAGGCCGAATATGATGAATCT
GGTCCTTCCATTGTTCATCGTAAATGCTTCTGAGTTGTGTGAAGACAAACCCAATGGTA
TATCTTCCCTTTATTTTCGAAACCGTGTCGTATTAGAGTTCTGTTTGGTTTTTTTTCAAGT
TTGGAGTAAAATGTTTGGAACTGTACTATGTTATAATATAGAGTTGGGTAATTTGTGATG
GGTGTATCTTAGGTTTTGCTGTTT

Figure S5. Nucleotide sequence of *HIS4* from *E. ulmoides.*

GGGAGGCTGGTCATTCAATCTTCGTCTCTCTCTCTGGTTTTCAAGCTCCGGCGAATTTT
TCCGAACAGCTAACGACGATGTCCGGCCGTGGAAAGGGAGGCAAGGGACTGGGAAA
GGGAGGTGCGAAGCGGCACCGTAAGGTCCTCCGGGACAACATCCAGGGAATCACGAA
GCCGGCGATTCGTCGTCTGGCTAGGAGAGGCGGAGTCAAGCGTATCAGCGGCCTCATC
TACGAGGAGACTCGCGGAGTCCTCAAGATCTTCCTCGAGAACGTGATCCGCGACGCCG
TCACCTACACCGAGCACGCCAGGAGGAAGACGGTGACCGCCATGGACGTCGTCTACG
CTCTGAAAAGGCAGGGAAGGACTCTCTATGGTTTCGGCGGTTGATCAAGTTAGCTTTA
GATCTGGATGTATATTACTATATTGTAAGGATTAGATTCTGTTAGTCTTTTGAATCTCCTA
GTGTTTGATTTTCAATTAGAATTCGATCTGTCTTACTACAATCTATCCGGTTGTTCTTCGT
TCAATTCTCTAATCACATGCTACTTTTTTGTTGCATCCATTTGTTTGCGGATTGTGATTTA
CTCTTATTACGTATGCTATGATTGTATTAGAAACTTCAAATTTCTTGTTGCCGTTTGAACA
ATCTTGGTATAATTTGATCTAAATCAACGACGGATTGGGTTTGATTTTTATCTAAAATAA
GGAAATGATTAATTTAGGCAAAATTGGATACTGTCCATGTTAGATATGGTGCACGCTGA
GTGTGCCATTAGGCATACACGGGGCCATTAGATTAGGCGCACACAGAGCATGATCGAG
TTGTTTTGAGTTCATTTTGAGATAGTATAGAGGACGACTGAGCTCTTAGGTTATCCTTTG
GCAGGAAGGAAGGAAAAGGAAAAGGGGATTTTTTTTTTTTTTTTCCTGATTCTGATTTT
TGCGGAGG

Figure S6. Nucleotide sequence of *HIS2B* from *E. ulmoides*.

TTGAAACGACAAGATCCATGAAATTAGAAAATTTTACACAAATTCAGGATTGTATTCCA
TATCTGTAATACACAAATTGGACAAAGAGGATCCAAAACAATCTTCAAAAGCAGAACA
CAAGCCAAAAGAGGCAGAAATAACCAAAAGGAACAAATATTTAACTACAACTAATATA
CTAACCCTAATCCTAGCAATTGGTATTCAGATATGCAGAGAATTGAAACACCGGATTCA
GCAATTACCAACAGTGTCTTCAGGAGCTCGTGAATTTCGTCACGGCCTTGGTCCCCTCA
GAAACAGCGTGCTTGGCCAATTCGCCGGGAAGCACCAATCTCACGGCGGTCTGGATCT
CCCGGGAGGTGATTGTCGGCTTCTTGTTGTACCTTGCGAGCCTCGACGACTCCTGAGC
AAGCTTCTCGAAGATATCGTTGATGAAGCTGTTCATGATGCCCATGGCCTTGCTCGAGA
TCCCGATGTCGGGGTGGACCTGCTTGAGGACCTTGAAGATGTAGATC

Figure S7. Nucleotide sequence of *TUB* from *E. ulmoides.*

GGTTCTAAATTTGACCCTCCTAACACAACTTATCAAAGAAGATGATGGTGTCTCCTTGG
ATGGGAAAAGGAACATTATATATTTTCCCAAAAAAACCCAAAGATTCATTCACAACCCA
AGACGACGACGCAAAGGGCACAAATTTAAAGCATAATATGCGAATAGAAACACCAATG
ACAAAATACACAACAAATCCAAAATACAACCATTTTCAGAGCACCAAGTTTCACAATT
TTTAAAAGCACCAAATCTCCCCCAACCTACAGACAAGATAGTAAGAAAAACTGGTAGT
CGAGGTTACAACCACAAGCCTATTACTTGCTATATGACAAACTAACACATAATAAAGCC
ATTCAAATTCACCATCACATTACCTTTTCAACCCCCCTCCCGTCCATACTCTCACTCCTG
ATACTCCTCTTCATCCTCGTACTCTCCCTCCTCATCGGCCGTCGCATCCTGATACTGCTG
ATACTCCGCCACCAAATCATTCATGTTGCTCTCCGCCTCAGTGAACTCCATCTCGTCCAT
CCCTTCTCCGGTGTACCAATGCAGGAAAGCCTTTCGCCGGAACATCGCCGTGAACTGC
TCGCTCACCCGCCGGAACATCTCCTGGATCGAGGTCGAGTTGCCGATGAAAGTCGAAG
CCATCTTCAATCCGGTCGGAGGAATGTCACAGACGGTGGATTTGACGTTGTTTGGGATC
CATTCGACGAAGTAGGAGGAGTTCTTGTTCTGGACGTTGATCATCTGCTCGTCGACTTC
CTTCGTGCTCATCTTACCGCGGAACATGGCGGAGGCCGTCAGGTAACGGCCGTGACGG
GGATCGGCGGCGCACATCATGTTTTTGGCGTCCCACATCTGCTGGGTGAGCTCTGGAA
CGGTAAGGGAACGGTATTGCTGCGACCCACGGGATGTGAGTGGAGCAAAACCGACCA
TGAAGAAGTGGAGTCTGGGGAAGGGGATGAGATTGACGGCGAGCTTCCGGAGATCGG
AGTTCAGTTGTCCGGGGAATCGCAAGCAGCAGGTAACGCCGGACATTGTTGCAGAAAT
CAAGTGGTTCAAATCGCCAAAGCTTGGAGTTGTGAGTTTCAGAGTACGGAAGCAAATA
TCGTAGAGAGCTTCGTTATCCAGAACCATACACTCGTCGGCGTTTTCAACCAGTTGATG
GACGGACAAAGTCGCGTTGTACGGCTCAACCACAGTGTCGGAGACCTTCGGAGATGG
AAACACGGAGAAGGTGAGCATCATCCGATCTGGATATTCCTCTCTGATTTTGGAAATCA
GAAGGGTACCCATTCCAGATCCAGTTCCTCCTCCAAGCGAATGACAGACCTGAAATCC
TTGAAGGCAATCACAATTTTCCGCCTCCTTGCGGACAACATCGAGAACCGAATCGATC
AACTCAGCGCCCTCGGTGTAATGTCCTTTCGCCCAATTGTTTCCGGCGCCGGATTGGCC
AAAAACAAAGTTATCAGGACGGAAAATCTGGCCGTAAGTTCCAGATCTGATGCTGTCC
ATCGTTCCAGGCTCAAGATCCATGAGCACAGCTCTAGGAACAAACCTTCCGCAACTCG
CTTCATTGTAATAGACATTGATTCTCTCAAGCTGCAGATCTGAGTCGCCGGTGTATCTTC
CGGTGGAATCGATGCCGTGTTCGGCACACACCACTTCCCAAAACTTGGATCCGATTTG
GTTGCCGCATTGGCCTCCCTGGATGTGAAGAATCTCTCTCATCTTTCCTTCTAATTGCGA
AGAAATTTACAGAAGAAAGGGTGGAAATAGAGAGAGAG

Figure S8. Nucleotide sequence of *UBC* from *E. ulmoides*.

GAATCCCCAATCTGGCATGCACCACAAAATACAAATTTACGCATATTCAAATGCGCTGC
AATTCCAGTTGTCAAGTAAAAAAGAAAGAGTTGAACAGACGACAATGAGCCTTACAA
TCTCTTCAAACTCTTCTTCCTGGCATCAATTTTCCTTTTGTCGGCCTGAACCTTTGCCAA
CCCAACTTCTTTCTTTGGCATCGCAACAACTTGTTCTGCTGACCCGCTAACTCCCGTTT
CGTTTGTTGAATTTCTAGCAACTGGCGGCTTATCCTGACCACTTCCTCCTTTCATGGTGT
TAGAAGAAGGTGCCAACGGACATGCTGATTGGACTACAGCACCACCCACTCCCGCATT
CTTTTGGTCCACACTATAAACTGGGGCATTTGTCTGATCGACATTCAAGACAGTAGTTG
ACTCCGAAATAGCTCCTGATTTGAACTTGGGTTTTGGCTTGAGAGCATGAATTCCAGTG
TAAAGCCTGTGTTTTTTAAGCCAAAGAAACATCCTCAAGTCACACAGTATCCAACAAC
TACAGATAACATGACGCAAAATTCTAAGTTTTTAACGTAGATGCACATTTCTTGAGAGA
AATCCAGTAGTCAACTATGACCCAAACCTGCGCTCCCATTTATCTTCAAACTAAGCTTAT
TTCATCCTCTTCAATCTTCCGAATAAACAAGAATATAGACATCAATAACAAAATATTTTC
AGAACGAGTACAAGTTTTCAGTATTTCATCAGTAGTTTTCAATATTTCATCAGTGCTTTG
TCGATTACGAAAATTATATTGCATGCAATCGGTTATATTGGTCATTTCTAATGATCTTAAC
AACCCGTTTCTAATAGGTTGACAGACCATCTTAAAAAATTTGGGCCTGTTTGGTCATCT
TCAGAAAACAGTTCTAGCTTTCTGGGGGAAAAAATGGCTTCACCTTTCGCAACACAAT
TTGAACACACTTCATAAAACAAAAAACCTTTAACAATGCTCAATCAATCGCCTCAACC
ACATTTTTCACAATTTTCATCACATCACCTCTAACTTTGAATTTGTTATCACATTATTATTT
TTTTTTTGGGTGGGTGGGTGAGTTTGGTTATGTAAACCAAACAACTTTCCAAAATTATT
TTTCAGAAATTATTTTCAGATTTACCAAAAAAATATTTCAAATAGGCCCTTTACACTCAA
CTTTCTCTTCTTAAATTTATTATTCTTTTTTCTACTTTTTCTTTTATTTATTTATATGAATAA
ATAAATATATATATAATTTAAAATGTACGAAATATAATTTAATTCGACTGTTTTGGAAGTT
GGGGTCAAATTCGATTTATCCATTTTTTTCTCAAATCAGTCCCAATACTTTACGAAAAAA
ACATAAGAAAATATCATATTTTATTATATTGACTAGAGATTCTCGGTGAATAAAGGGAATA
TGCT

Figure S9. Nucleotide sequence of *UBA52* from *E. ulmoides.*

CTGCGCTCCCGTCCCCATATAAACAACCTCAAAGCAAAACCCTCGTAGCCCCCGAGCG
TAGCAGACAGAGAAGGACCAAGAAAATGCAGATCTTCGTGAAGACCTTAACTGGGAA
GACTATCACCCTCGAGGTTGAATCCAGCGACACCATCGACAATGTTAAGGCCAAGATT
CAGGACAAGGAAGGTATTCCGCCGGACCAGCAGAGGCTGATCTTTGCAGGGAAGCAG
CTCGAGGATGGCCGTACCCTCGCAGATTACAACATCCAGAAAGAGTCAACTCTTCATC
TTGTTCTGAGGCTTCGAGGTGGCATTATTGAACCATCTCTGATGGCATTGGCCAGGAAA
TACAACCAGGAGAAAATGATCTGCCGCAAATGTTATGCACGGTTGCACCCACGTGCTG
TCAACTGCAGGAAAAAGAAGTGTGGACACAGCAACCAGCTGAGGCCAAAGAAGAAG
ATCAAGTAGAGTAGTTGGTGTGAGGAAAAATTGCTAACCATTGTAGTGGGTCGCTGGA
TGTCTTATCTGAACGATGGTAGCATCAAACAGTTCGCTAGTAGAATTCTAGAAATCCGA
CATGTAGTACAGTGTTACTTAATATGCTTTGAACTTTTTGGGCGTTGAATATGCCGTGAC
GTTTTGAGTCATTTTTTTTGGTGATGAAATCAAACCTATGCTTTTGTTTAGTTTTATGTGG
GTTCTGTTGCTTAATTATGTTTGTTTGGTCCTTGAGCTCTTCAATTTTGAGGCGTTGGAA
TGAAAGACTGTCTGACTGTGTAAGACGGATTTTGTGGGAA

Figure S10. Nucleotide sequence of *UBA80* from *E. ulmoides.*

GGAACTCCGCCATCAAAATCCAAAATTCATATCAAAAGCAAGCAACAGGAAAGGAAA
AAAACAGAATTGAAGATCCAGAACATGTTTCATCAATGGAGGAACATCTGAAAACTAG
AAATTTTAAGCAGAAATTGAATCTCTAATCGGCGCCCGCCTTATTGTAAACATAGGTGA
GGCCACACTTACCACAGTAGTGACGATCAAAGTGATTCGCCATGAAAGTCCCTGCACC
GCACTCGGCGTTCGGGCACTCCTTCCTCAGCCTCTGCACCTTCCCCGAATCATCCACCT
TGTAGAACTGAAGCACGGCGAGCTTCACCTTCTTGTGCTTGTGCTTGATCTTCTTCGGC
TTGGTGTAGGTCTTCTTCTTCCTCTTCTTCGCACCGCCGCGGAGCCTGAGGACCAGGTG
GAGGGTGGATTCCTTCTGGATGTTGTAGTCGGCTAGGGTTCTGCCGTCCTCGAGCTGCT
TTCCGGCGAAGATGAGGCGCTGCTGGTCCGGCGGGATTCCTTCCTTGTCTTGGATCTTG
GCCTTGACGTTATCG

Figure S11. Nucleotide sequence of *UBC E2* from *E. ulmoides.*

GGAGTGTGAATTTTTCTATGCAGACTCGAACAAATGCTCTCTCTTTCGAATAGATAAATAGTTTTTCAGA
TGAAAACAAAGTCGTTTCAGCTCCAAAGAATCGTTTGCTGAGGGCGATCGCAGCGTCTTTTCCTCATT
TTCTGTGTCTCCGATCGATGGGGTCGCCGCCGATGGCCACATACATCCTCCCGAATTCTAAGAAAAGGC
TGTCAACTGAGGGAACCTCTCCGTTTTGGGAGCCGGATGTTGAGGAAATTGCGCCGCCAATATCATGG
AATTCGATGTCGAAGTCGAAGTCGCTTAAACAAAAGAGGGAATTTTTTCACGAAGTAATTGATGTTGA
CATGGATGAAGATTGTTTTGATGTTATGTATACTGATGGGAAGATTGACTCAAATAATAAGGGAAAGGA
AGTTTTGGTCGATTCTTTGTTCAATCCTAGCAGTACAACTACAACAAAGCAGGAATCGGTGGAGGGGT
TTCCGCATTCTCTTAAGGCCTTTAATCCAGGATTACATAGTATGGTTAATCCAGATGGTACAAGTTCCAA
TTGTTCTTATGGTGATAGTATTGATATCAATATGAATCATGATGATTCCTCGTTGGATGATGACCCCTACC
ATCGTTTGCAATCTTATTTTGATAACATGGATGTTCCTCCTGGTGTAGAGGCTCCAGTGCCTTGGTTGCT
AGACCCTTGTTCCAATAGTATGACTCTTTCTACCACTAGCAGTTCAACCCCTTTAAGTTTAGGGATTGAA
TCAGATGCTGCAGGAATCCCCCCTGTTCCAGAATTTTCTCACTCCACGTGGACGCCAAATCCTACTCAG
AGAATTTTGGAGTCAACCTCAAGCACTGATTTGGGTTTTCAAACACCACCATATGGACTGGAAAATCT
TGACAAGTCTGATGTCTCTTCATTTCAGTTGTTTGTGAAACCTTGTGGTCCTTCTCAAAGGAAAAAGA
AATCAACTGCTTCAGGCAGTTCCAGTTTTTCGAATTTTCATAAGAGAGTTACCGGTAGTTTTCCTGTGG
AGGAACCCTTCAAATCTCGACACGGTGATTCTACCTTCGTAAAAAGGAAGCATCATTTTCCAACCAGT
ACATCATCTTATAGCTCAAATGGCCATGCTGGTGGGTTTTCGTTTTCAAATGGCACAAACAAGCAAGTT
GGTAATGGTGGTTCAAGTTTTTATATGCAAATGGATGTAAATAACGTTCTTTCTGGAGTAGAAGAAGAC
TCCATCCCTGATTTGGGGGGTTTCCTGGAGTTTCCCATGACTGTGATGGAGCAAGATCCTACGAAGTTC
TTTGATGCATCGGGTGCTGGAAACATGTTTCCCGGGGAGATGGCTTATAGCTCCTGGGACTCTGATCCT
TATGAAACTCAACAATGTGCGACTGCTTCAGCAAGTGTGACGATGACAGAGGATGAAATTTTCGAGAA
GTTTGATGCATTTAAGAAGTTTGACACTGTTCAAGATCATTCAGATCATCACTATTCAAACCCTGGTTCT
TCAGCCGGGCAGGTAATTGATATAAAAAAACATATATCATATACTCCCACCTTCCCCAGAAAACTATTAG
GTTGGACTCATATATCTAAGTTATTTTACTATGATTTAATTGCCTTCAATTATAAGTTGTGTTTCATCGATT
CAAGTAAATGGATTCACCTCCCGTTTGCTGTGCTGAAGAGAATATAAAGAATTGATTACCACAGATGTT
TTACGGTGTTTGGAAATTCATTGCATTAGGCTCCTTGTTTATTTTCTGTTGGCAGCCATCAAAGAATTGG
GCGAAGAAAATACAGGAGGAATGGAAGATCCTAGAGAAGGATTTGCCTGATACGATATTTGTTAGGGT
TTATGAATCCAGGATGGATCTCTTGAGGGCTGTAATTGTAGGAGCAGAGGGGACACCATACCATGATGG
TCTTTTCTTCTTTGACGTTTTTTTCCCCACCACCTATCCTAATGTCCCGCCGCATGTGTGGTACCACTCT
GGTGGTGTTCGAATCAACCCAAATTTGTACAACAATGGAAAAGTTTGCCTGAGTCTCCTTAACACCTG
GAGTGGCAACCACCGAGAGAAGTGGATCCCTGGTGTGTCCACCATGCTCCAAGTTCTGGTCTCTATAC
AAGGGTTGATTTTGAATGCAAAGCCCTTTTTCAACGAACCTGGATATGCGAGTATGGCTGGGTCAGTG
AGTGGAGAAAAGAGATCCCTGCAGTACAATGAGTCTACGTTTATCCTATCACTGAAAACTATGGTCTAC
TCTATGAGGAAACCACCAAAGCATTTTGAGGACCTTGTTGTCGGGCATTTCCACAAGCGAGCTCATGA
TATTCTAGTGGCATGTAAAGCATACATGGACGGGGCTCAAGTAGGGTGCCTTGTTAAAGGTGGTATTCA
GGATGTTGACGAGGGCGATAAGAGCTGCTCACATTCTTTTAAGACATCGTTAATGGGTTTCACTAAAAC
TCTTGTCAAAGCGTTTGCACGAATTGGAGTGAAGGATTGTGAGAAATTCCTTGCGCCAGTACAGAAAT
CATGACAAGGAAAACAGGCACTATGATATAACAGGGTTGAGTCCTTTATTTTATTTTATTTTTTTGGGCA
AAATTAAGGTTTATTAGACACAAGAACACAATTTCTGCAGGTAATTAGTTAAATTCAACCATTGTAGTG
GACTTTGATGATTTCGGGATGC

Figure S12. Amplification of qRT-PCR specific primers.

Amplified fragments of 11 candidate reference genes in *E. ulmoides* were separated by 1.2% agarose gel electrophoresis. Lane 1：Marker 50; Lane 2: *TUA*; Lane 3: *18S rRNA*; Lane 4: *ACT97*; Lane 5: *ACT*; Lane 6: *HIS4*; Lane 7: *HIS2B*; Lane 8: *TUB*; Lane 9: *UBC*; Lane 10: *UBA52*; Lane 11: *UBA80*; Lane 12: *UBC E2*.

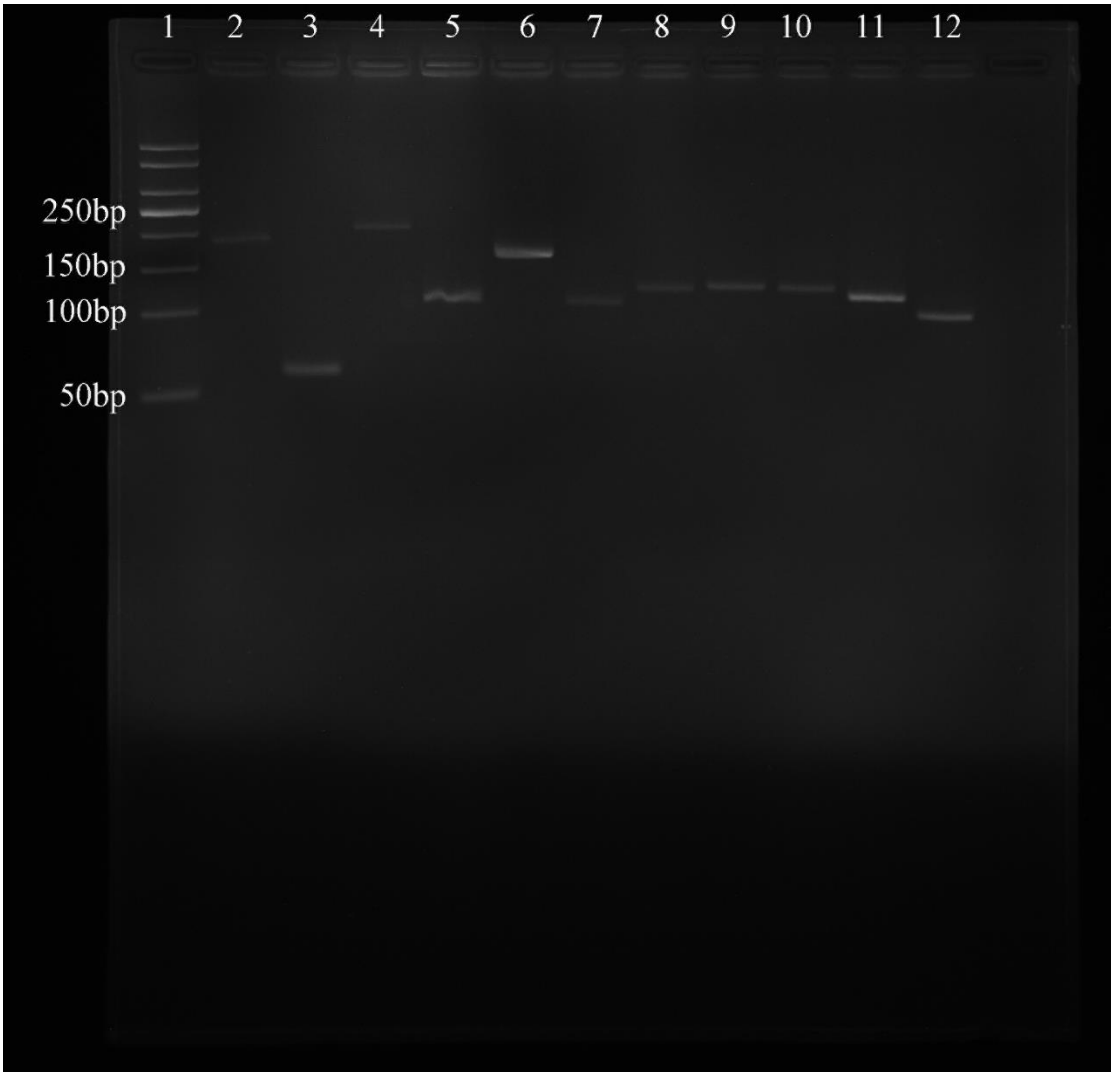

Figure S13. Melting curve for 11 candidate reference genes.

The melting curve for *TUA*, *18S rRNA*, *ACT97*, *ACT*, *HIS4*, *HIS2B*, *UBA80*, *UBA52*, *UBC*, *UBC E2*, *TUB* with single peak obtained from three technical replicates of different cDNA with no template control.

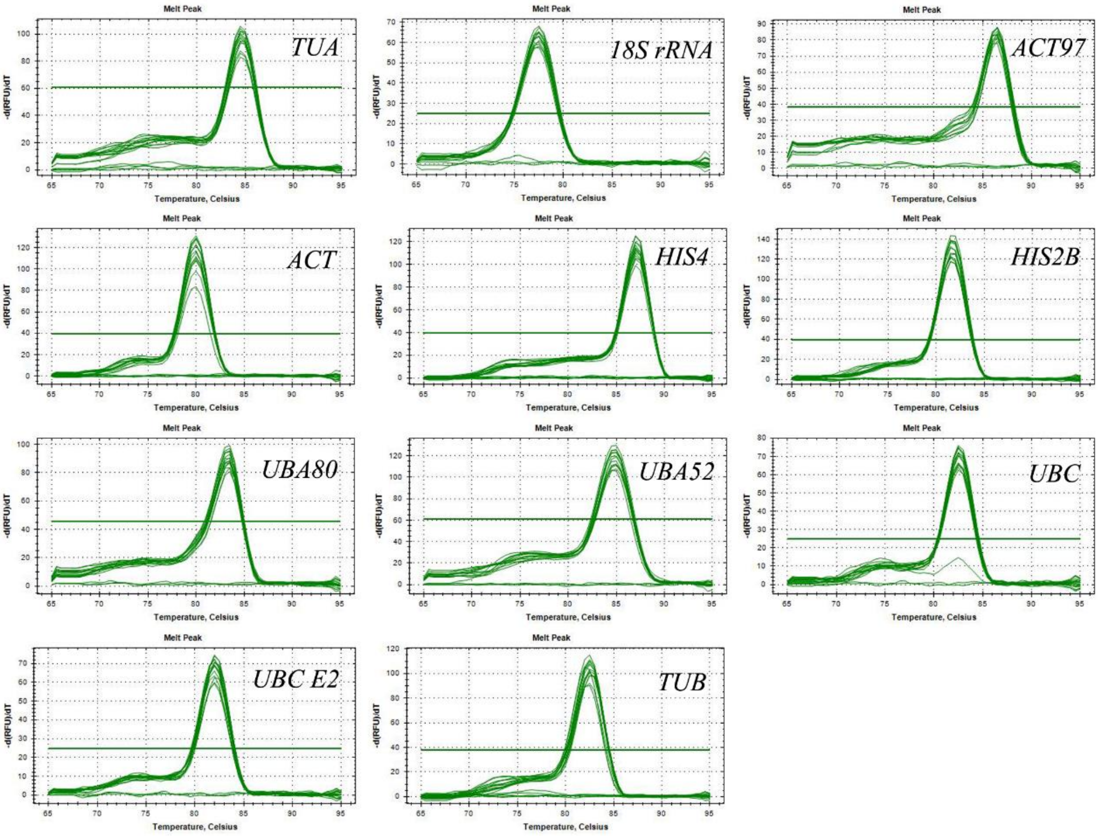

Figure S14. Standard curves of each candidate reference genes.

Standard curves of *TUA*, *18S rRNA*, *ACT97*, *ACT*, *HIS4*, *HIS2B*, *UBA80*, *UBA52*, *UBC*, *UBC E2*, *TUB*. The linear correlation (R^2) and PCR efficiencies (%E = ($10^{[-1/slope]}$-1)×100%) were calculated from the standard curve.

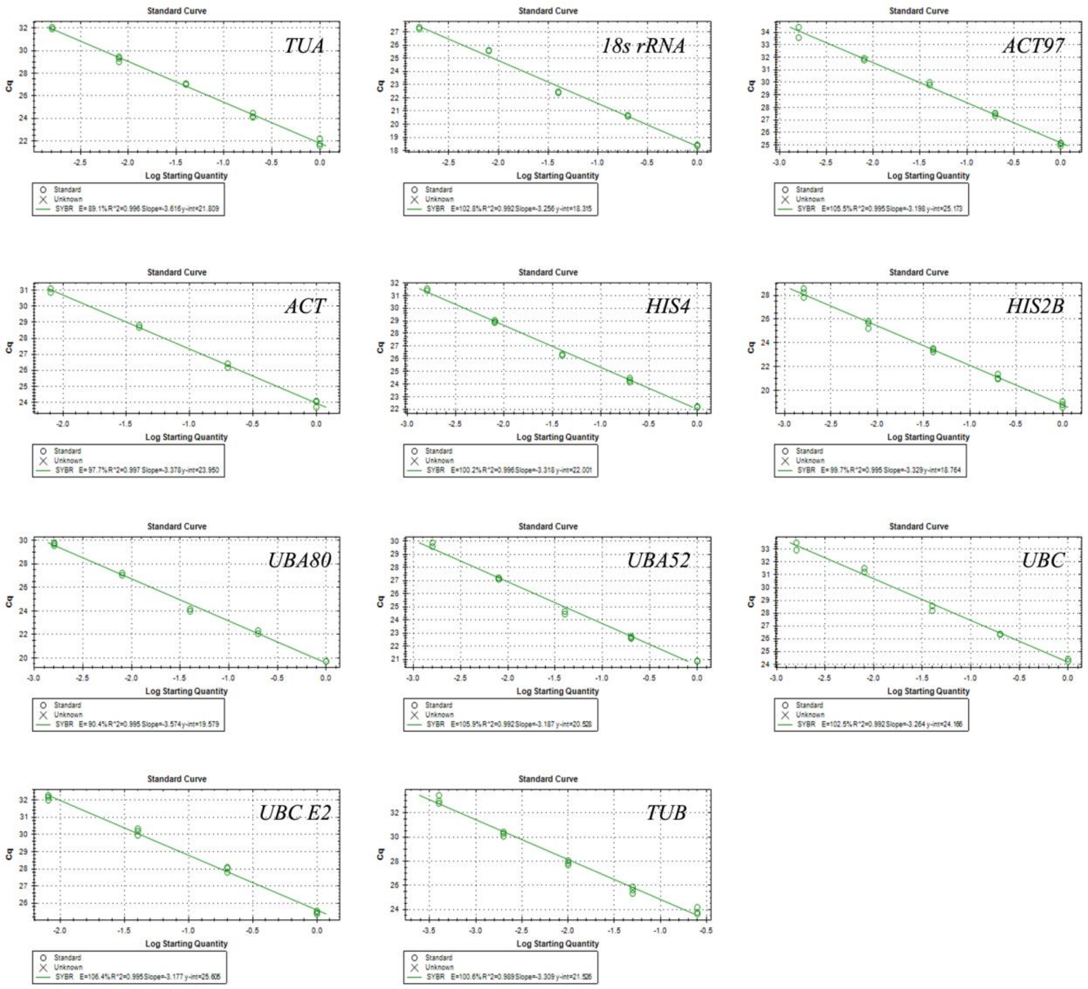

Figure S15. The average expression stability values (*M*) of the candidate reference genes analyzed by geNorm.

Expression stability was evaluated in samples from *E. ulmoides* submitted to total samples, total natural growth samples, total abiotic stresses samples, different varieties, different periods, different tissues, drought stress, cold stress, salinity stress. The most stable reference genes were measured during stepwise exclusion of the least stable reference genes. The least stable genes with highest *M* values, and most stable genes with lowest *M* values.

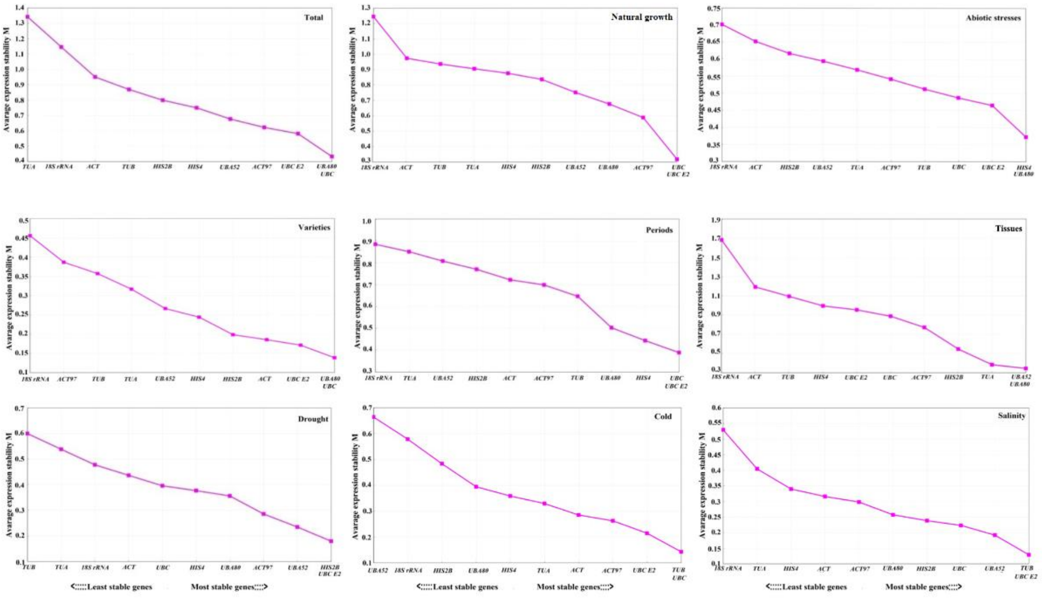

Table S1. Primer sequences for cloning of 11 reference genes from *E. ulmoides*.

Gene symbol	Primer sequence (5'-3') forward/reverse	Amplicon length (bp)
TUA	GGAGCGTCTCTCTGTTGATTATGGT<br>ACAGTGAATGAATTGTTGAAAGGTCC	1171
18S rRNA	AAGCTCGTAGTTGAACCTTGGGC<br>CTCGCTCCCTCCTCTGCCTA	432
ACT97	AGATGATGCTCCACGGGCTGT<br>CCAAACAGAACTCTAATACGACACGGTTTCG	1137
ACT	AATTATATGGGACCGTCAAACATGC<br>AAACAGCAAAACCTAAGATACACCC	1314
HIS4	GGGAGGCTGGTCATTCAATCTTC<br>CCTCCGCAA AAATCAGAATCAGGAA	951
HIS2B	TTGAAACGACAAGATCCATGAAAT<br>GATCTACATCTTCAAGGTCCTCAAG	515
TUB	GGTTCTAAATTTGACCCTCCTAACACAAC<br>CTCTCTCTCTATTTCCACCCTTTCTTCTG	1793
UBC	GAATCCCCAATCTGGCATGCACC<br>AGCATATTCCCTTTATTCACCGAGAATCTC	1434
UBA52	CTGCGCTCCCGTCCCCATATAAA<br>TTCCCACAAAATCCGTCTTACACAGTCAGAC	799
UBA80	GGAACTCCGCCATCAAAATCCAA<br>CGATAACGTCAAGGCCAAGATCCA	542
UBC E2	GGAGTGTGAATTTTTCTATGCAGACTCG<br>GCATCCCGAAATCATCAAAGTCCACT	2713

Table S2. Raw Cq values of 11 reference genes in *E. ulmoides.*

	18S rRNA	*ACT*	*ACT97*	*HIS2B*	*HIS4*	*TUA*	*TUB*	*UBA52*	*UBA80*	*UBC*	*UBC E2*
variety1-1	25.99	24.49	25.41	24.20	19.65	21.09	23.60	21.36	21.41	23.91	23.12
variety1-2	26.16	25.19	25.76	25.55	20.71	22.24	24.60	21.95	22.11	24.72	23.09
variety1-3	26.54	23.82	25.27	24.39	19.70	21.06	23.70	20.74	21.11	23.74	22.63
variety2-1	25.44	24.40	24.92	24.53	20.05	21.30	24.06	21.44	21.50	24.29	23.09
variety2-2	25.94	25.05	25.63	25.36	20.83	22.02	24.87	21.28	21.70	24.79	23.20
variety2-3	25.24	25.29	24.98	26.70	21.34	23.16	24.55	22.78	22.32	24.63	23.49
variety3-1	26.25	24.06	24.60	23.88	19.31	20.42	23.54	20.76	20.90	23.34	22.61
variety3-2	28.42	24.46	25.43	24.41	20.09	21.41	24.55	20.61	20.61	24.02	22.86
variety3-3	24.48	23.54	24.92	25.21	20.67	21.61	24.05	21.33	21.35	24.12	22.89
variety4-1	26.28	24.04	24.42	24.62	20.49	20.66	23.10	22.05	21.52	24.12	22.94
variety4-2	27.42	24.25	24.69	25.09	20.84	20.75	23.34	21.95	21.48	24.33	22.89
variety4-3	25.78	24.32	24.60	25.05	21.04	21.13	23.54	21.95	21.42	24.54	22.91
Period1-1	24.50	25.26	25.45	23.99	19.59	21.63	26.51	21.46	21.49	24.09	23.03
Period1-2	24.50	25.08	25.95	24.22	20.84	21.72	25.46	22.57	22.72	25.05	23.70
Period1-3	24.50	25.56	26.46	24.45	20.49	21.81	25.39	21.91	22.03	24.45	22.98
Period2-1	25.65	24.43	25.04	24.39	20.15	21.48	24.65	21.50	21.43	24.27	23.06
Period2-2	25.51	23.50	25.13	23.85	19.45	24.18	24.07	20.57	20.99	23.75	22.33
Period2-3	25.38	25.75	25.22	24.12	21.58	23.50	24.70	22.63	22.59	24.66	22.89
Period3-1	24.18	24.21	25.58	25.08	20.79	21.13	24.19	23.20	22.34	25.32	24.01
Period3-2	23.68	24.10	25.49	24.11	20.24	24.07	24.36	22.84	22.46	25.06	24.18
Period3-3	23.17	23.33	25.67	24.59	20.52	19.03	24.28	23.02	22.40	25.19	24.09
Period4-1	24.65	24.46	25.70	24.20	20.16	21.52	25.38	22.91	22.47	25.08	23.15
Period4-2	24.43	26.99	25.96	24.24	20.08	22.49	25.06	23.77	23.33	25.34	23.25
Period4-3	24.22	25.45	26.21	24.16	20.24	23.45	24.73	24.64	24.18	25.60	23.36
Period5-1	23.52	24.21	23.79	26.29	21.39	24.63	25.44	24.05	22.99	25.14	24.18
Period5-2	24.12	23.77	24.46	25.67	20.38	25.48	23.42	22.84	22.19	24.48	23.35
Period5-3	24.72	25.36	25.14	25.98	20.89	25.32	24.85	24.55	22.59	26.08	24.49
Bark-1	21.80	23.84	25.49	25.63	22.27	22.94	23.02	22.38	21.88	23.92	22.91
Bark-2	20.61	22.66	25.24	24.96	21.97	22.30	23.37	21.99	21.35	23.48	22.46
Bark-3	21.09	22.92	25.31	25.28	22.28	22.54	23.22	22.12	21.51	23.58	22.60
Branch-1	25.68	23.65	23.50	25.67	21.32	20.84	22.24	21.26	20.51	23.82	22.10
Branch-2	26.79	22.87	23.16	24.54	21.34	20.43	22.27	21.11	20.35	23.35	22.12
Branch-3	26.50	23.80	23.65	24.99	21.33	21.31	22.23	21.44	20.79	23.83	21.86
Leaf-1	25.91	24.56	25.19	24.80	20.41	22.09	24.06	21.52	21.27	24.31	23.03
Leaf-2	25.74	24.88	25.66	24.96	20.51	21.81	24.49	21.44	21.72	24.13	23.05
Leaf-3	25.95	25.88	25.57	24.90	20.45	21.90	24.34	21.45	21.54	24.20	23.04
Root-1	22.21	25.08	28.21	29.04	24.23	26.95	29.17	26.53	26.10	26.36	24.53
Root-2	22.51	25.67	27.72	28.88	23.98	26.65	29.24	26.54	25.92	27.02	24.63
Root-3	22.36	25.38	27.22	28.96	24.03	27.25	29.21	26.52	25.74	25.76	24.58
Cold0-1	25.72	25.02	26.32	28.57	23.24	27.36	25.06	23.06	23.99	26.67	24.84

Cold0-2	25.65	24.92	26.28	28.63	23.2	27.41	25.02	23.11	23.92	26.71	24.75
Cold0-3	25.79	25.12	26.36	28.51	23.28	27.31	25.10	23.01	24.06	26.63	24.93
Cold2-1	22.81	24.99	26.54	27.18	22.26	26.93	24.86	24.91	22.55	26.23	24.32
Cold2-2	22.84	24.93	26.57	27.21	22.33	27.06	24.90	24.93	22.48	26.30	24.27
Cold2-3	22.87	24.87	26.60	27.24	22.38	27.19	24.94	24.95	22.41	26.37	24.22
Cold6-1	25.03	24.94	26.35	27.40	22.76	27.30	24.67	23.23	22.99	26.04	24.64
Cold6-2	25.11	25.02	26.32	27.47	22.71	27.25	24.61	23.25	23.00	25.95	24.60
Cold6-3	24.95	24.86	26.38	27.33	22.81	27.35	24.72	23.21	22.98	26.13	24.68
Cold9-1	24.22	24.37	25.86	26.59	22.16	27.08	24.73	23.85	22.91	26.21	24.38
Cold9-2	24.32	24.49	25.96	26.52	22.24	26.96	24.89	23.81	23.07	26.13	24.54
Cold9-3	24.27	24.43	25.91	26.66	22.08	27.20	24.81	23.83	22.99	26.17	24.46
Cold12-1	24.05	24.28	26.15	25.83	21.82	26.25	24.67	23.65	22.49	26.34	24.71
Cold12-2	23.96	24.35	26.08	25.79	21.89	26.21	24.69	23.58	22.57	26.39	24.74
Cold12-3	24.14	24.21	26.23	25.75	21.75	26.29	24.65	23.72	22.41	26.29	24.68
Drought0-1	24.41	24.09	26.20	27.81	23.19	26.42	24.39	24.25	23.25	25.61	25.06
Drought0-2	24.35	24.16	26.13	27.74	23.15	26.44	24.35	24.28	23.16	25.66	25.00
Drought0-3	24.47	24.02	26.27	27.88	23.23	26.40	24.43	24.22	23.34	25.56	25.12
Drought2-1	24.26	24.16	26.01	28.09	22.81	26.57	23.57	24.05	22.96	25.54	24.91
Drought2-2	24.22	24.20	25.95	28.17	22.77	26.60	23.55	24.07	22.92	25.59	24.88
Drought2-3	24.30	24.12	26.07	28.01	22.85	26.54	23.59	24.03	23.00	25.49	24.94
Drought6-1	24.04	22.64	25.40	27.43	22.67	27.24	24.23	23.62	22.92	25.73	24.34
Drought6-2	24.08	22.69	25.31	27.48	22.63	27.18	24.19	23.64	22.86	25.75	24.31
Drought6-3	24.00	22.59	25.49	27.38	22.71	27.30	24.27	23.60	22.98	25.71	24.37
Drought9-1	24.32	22.49	24.89	26.90	22.73	26.69	24.80	23.21	22.49	25.45	24.01
Drought9-2	24.28	22.53	24.81	26.95	22.70	26.76	24.84	23.24	22.45	25.50	23.96
Drought9-3	24.36	22.44	24.97	26.85	22.76	26.62	24.76	23.18	22.53	25.40	24.06
Drought12-1	25.01	23.00	25.53	26.68	22.89	27.63	25.31	23.35	23.16	25.66	23.58
Drought12-2	25.00	22.96	25.50	26.72	22.91	27.72	25.36	23.32	23.11	26.70	23.55
Drought12-3	25.02	23.04	25.56	26.63	22.87	27.54	25.26	23.38	23.21	25.62	23.61
Salinity0-1	23.24	24.10	26.28	27.31	23.18	26.42	24.89	23.53	23.01	25.98	24.36
Salinity0-2	23.18	24.17	26.22	27.25	23.21	26.32	24.94	23.51	22.95	25.92	24.40
Salinity0-3	23.30	24.03	26.34	27.37	23.15	26.52	24.84	23.55	23.07	26.04	24.32
Salinity2-1	24.44	24.64	26.67	27.01	22.44	27.28	24.29	23.07	22.42	25.74	24.06
Salinity2-2	24.41	24.67	26.63	27.07	22.47	27.25	24.31	23.10	22.45	25.77	24.08
Salinity2-3	24.47	24.61	26.71	26.95	22.41	27.31	24.27	23.04	22.39	25.71	24.04
Salinity6-1	27.02	24.93	26.95	28.53	23.27	28.84	25.29	24.27	23.61	26.80	25.04
Salinity6-2	26.99	24.96	26.91	28.56	23.22	28.87	25.24	24.29	23.59	26.84	25.07
Salinity6-3	27.05	24.90	26.99	28.50	23.32	28.81	25.34	24.25	23.63	26.76	25.01
Salinity9-1	24.10	24.52	26.59	27.43	22.24	27.26	24.32	23.50	23.30	26.30	23.89
Salinity9-2	24.08	24.47	26.55	27.45	22.27	27.30	24.37	23.46	23.32	26.34	23.87
Salinity9-3	24.12	24.57	26.63	27.41	22.21	27.22	24.29	23.54	23.28	26.26	23.91
Salinity12-1	24.51	24.58	26.76	27.37	23.03	27.32	24.51	23.59	23.21	26.16	24.23
Salinity12-2	24.53	24.66	26.71	27.33	22.98	27.28	24.48	23.63	23.17	26.24	24.27
Salinity12-3	24.49	24.62	26.81	27.35	23.08	27.30	24.54	23.61	23.25	26.20	24.20

Table S3. Data statistics of Cq values of candidate reference genes in all samples.

	N Total	Median	Mean	Std Dev	Minimum	Maximum
18S rRNA	84	24.45	24.64	1.36	20.61	28.42
ACT	84	24.41	24.33	0.88	22.44	26.99
ACT97	84	25.81	25.78	0.89	23.16	28.21
HIS2B	84	26.65	26.32	1.50	23.85	29.04
HIS4	84	22.24	21.88	1.24	19.31	24.23
TUA	84	26.41	24.95	2.71	19.03	28.87
TUB	84	24.55	24.60	1.17	22.23	29.24
UBA52	84	23.22	23.10	1.28	20.57	26.54
UBA80	84	22.56	22.60	1.08	20.35	26.10
UBC	84	25.59	25.36	0.99	23.34	27.02
UBC E2	84	24.05	23.86	0.83	21.86	25.12
